# MIF Deficiency Modulates Gut Microbiota Composition and Promotes Colitis-Associated Colorectal Cancer in a Murine Model

**DOI:** 10.3390/cimb48070712

**Published:** 2026-07-13

**Authors:** Sonia H. Navia, Oscar Illescas, Miguel Atl Silva-Magaña, Imelda Juárez-Avelar, Mariana Terrazas-Rodriguez, Felipe Vaca-Paniagua, Clara Estela Díaz Velásquez, Libia Vega, Luis I. Terrazas, Miriam Rodríguez-Sosa

**Affiliations:** 1 Laboratorio de Inmunidad Innata, Unidad de Investigación en Biomedicina (UBIMED), Facultad de Estudios Superiores Iztacala (FES-I), Universidad Nacional Autónoma de México (UNAM), Av. de los Barrios 1, Los Reyes Iztacala, Tlalnepantla 54090, Estado de México, Mexico; so.navia@ciencias.unam.mx (S.H.N.); imelda_juarez@yahoo.com (I.J.-A.); marianaterrazas@yahoo.com (M.T.-R.); felipe.vaca@gmail.com (F.V.-P.); cdiazvelasquez@aol.com (C.E.D.V.); literrazas@unam.mx (L.I.T.); 2 Genetic Epidemiology and Pharmacogenomics Unit, Department of Research, Fondazione IRCCS Istituto Nazionale dei Tumori (INT), 20133 Milan, Italy; oscarillescas.pomposo@istitutotumori.mi.it; 3 Programa de Doctorado en Ciencias Bioquímicas, Universidad Nacional Autónoma de México (UNAM), Ciudad de México 04510, Mexico; miguelatlsm@ciencias.unam.mx; 4 División de Genómica Computacional, Instituto Nacional de Medicina Genómica, Periférico Sur, 14610 Ciudad de México, Mexico; 5 Programa de Doctorado en Ciencias Biológicas, Universidad Nacional Autónoma de México (UNAM), Ciudad de México 04510, Mexico; 6 Laboratorio Nacional en Salud, Facultad de Estudios Superiores Iztacala (FES-I), Universidad Nacional Autónoma de México (UNAM), Av. de los Barrios 1, Los Reyes Iztacala, Tlalnepantla 54090, Estado de México, Mexico; 7 Departamento de Toxicología, Centro de Investigación y de Estudios Avanzados del Instituto Politécnico Nacional, Av. IPN 2508, Zacatenco, 07360 Ciudad de México, Mexico; lvega@cinvestav.mx

**Keywords:** fecal microbiota transplantation, dysbiosis, host–microbiome interactions, tumor microenvironment, immune–microbiota crosstalk, microbial biomarkers

## Abstract

Intestinal dysbiosis is a hallmark of both inflammatory bowel conditions and colorectal cancer, yet the mechanisms by which inflammatory mediators alter microbial communities and may contribute to tumor development remain poorly understood. Macrophage migration inhibitory factor (MIF) is a proinflammatory cytokine involved in innate immunity and the progression of inflammatory and neoplastic disorders. In this study, sequencing of the microbial 16S rRNA gene was performed to characterize the fecal microbiota profiles of wild-type (WT) and MIF-knockout (MIF-KO) BALB/c mice subjected to AOM/DSS-induced colitis-associated colorectal cancer (CAC). CAC induction resulted in marked microbial shifts, including increases in Muribaculaceae and Bacteroidota, in both WT and MIF-KO mice. Notably, MIF-KO CAC mice developed more severe disease compared with WT CAC mice. Furthermore, FMT experiments revealed that the fecal microbiota from MIF-KO donors was associated with increased tumor burden in WT recipients under CAC-inducing conditions compared with that in recipients colonized with WT-derived microbiota. Together, these findings suggest that MIF deficiency is associated with gut microbiota remodeling during CAC and support a potential relationship between the MIF-dependent host context, microbial composition and colorectal cancer severity.

## 1. Introduction

Colorectal cancer is the third most common malignancy and the third leading cause of cancer-related mortality worldwide [1]. Patients with inflammatory bowel disease (IBD), such as ulcerative colitis or Crohn’s disease, have an increased risk of developing colorectal cancer (18% and 8.3%, respectively) [2]. The pathogenesis of IBD and its progression to colorectal cancer is multifactorial and involves genetic predisposition, age, environmental factors, and chronic inflammation [3]. Emerging evidence indicates that alterations in the gut microbiota composition also critically influence the pathogenesis and progression from IBD to colorectal cancer [4].

The intestine is populated by commensal microorganisms primarily located in the distal small intestine and colon. These microorganisms interact with local epithelial cells, affecting mucosal permeability, nutrient absorption, and local immune responses [5]. Certain dietary habits or antibiotic use can induce shifts in bacterial populations and their metabolic activities; such dysbiosis of the bacterial community may facilitate intestinal colonization by pathobionts and pathogens [6].

Dysbiosis has been documented in both patients and animal models of IBD and colorectal cancer, with bacterial communities that are significantly distinct from those observed in healthy subjects [7]. In colorectal cancer patients, *Streptococcus bovis* is associated with epithelial damage, whereas *Fusobacterium nucleatum* is specifically linked to the adenoma-to-carcinoma transition. In contrast, beneficial bacteria such as *Clostridium butyricum* and *Streptococcus thermophilus* are significantly depleted [8].

Notably, microbiota modulation through fecal microbiota transplantation (FMT) from healthy donors to IBD patients has been shown to improve disease outcomes [9,10]. Furthermore, recent clinical and murine studies have demonstrated that the gut microbiota plays a crucial role in anastomotic healing. This is critical because anastomotic leakage is a major complication that increases mortality and colorectal cancer recurrence [11].

Chronic colon inflammation disrupts the mucosal barrier, leading to adenoma formation. These alterations facilitate microbial interactions with immune cells. Bacteria are detected by immune cells via pattern recognition receptors (PRRs), such as Toll-like receptors (TLRs), triggering the release of inflammatory mediators and a respiratory burst. This cascade exacerbates inflammation and heightens the risk of colorectal cancer development [5,12]. However, whether resident gut bacteria are the cause or the consequence of inflammation and subsequent colorectal cancer development remains unclear; similarly, the relationship between inflammatory mediators and the dysbiosis involved in this pathological process remains poorly understood.

Macrophage migration inhibitory factor (MIF) is a proinflammatory cytokine essential for robust innate immunity, as it is required for optimal TLR4 expression and adequate macrophage activation [13,14]. MIF-deficient mice are susceptible to a broad spectrum of infectious diseases [15,16]. In the context of intestinal inflammation, such as IBD, MIF overexpression has detrimental effects [17].

In a murine model of colitis-associated colorectal cancer (CAC), the administration of the carcinogen azoxymethane (AOM) combined with mucosal barrier disruption via dextran sulfate sodium (DSS) induces chronic inflammation and subsequent CAC development [18]. Previously, we used this model and reported that compared with WT mice, MIF-KO mice have greater tumor burdens and larger and more aggressive lesions. The differences in MIF expression between intratumoral cell populations suggest that MIF plays an important role in cellular differentiation. Interestingly, compared with those of WT mice, the intestinal epithelium of MIF-KO mice presented higher relative gene expression of IL-17 transcripts and fewer tumor-associated macrophages (TAMs). These findings suggest that MIF is involved in the immune context of early CAC development, including macrophage-associated responses and IL-17-related inflammation [19]. Because both IL-17 signaling and macrophage activity are closely connected with mucosal immunity, epithelial barrier regulation, and host–microbiota interactions, these observations raise the possibility that MIF deficiency may also be associated with changes in the intestinal microbial environment during CAC. However, whether such microbial changes are linked to altered immune responses or arise secondarily during inflammation-associated tumor progression remains unclear.

We hypothesize that MIF deficiency alters host microbiota homeostasis during AOM/DSS-induced injury, increasing susceptibility to CAC. To test this hypothesis, we utilized the AOM/DSS-induced CAC model and performed 16S rRNA sequencing of the fecal microbiota from WT and MIF-KO mice to analyze the role of MIF deficiency in microbial communities and its impact on CAC progression. We further evaluated this effect through FMT experiments in which MIF-KO donors were transferred into WT mice. Our results highlight a previously unrecognized role for MIF in regulating the establishment of a microbiota that is a determinant of CAC development and malignancy.

## 2. Materials and Methods

### 2.1. Animals

Female 8- to 10-week-old WT and *Mif*^−/−^ (MIF-KO) mice on a BALB/c genetic background were used. MIF-KO mice were generated as previously described and backcrossed for more than ten generations [20]. The animals were allocated to the experimental groups according to their genotype and treatment conditions. Both WT and MIF-KO mice were maintained under pathogen-free conditions, adhering to the Institutional and Mexican Guidelines for Animal Care and Maintenance (NOM-062-Z00-1999, 2002) and the U.S. National Institutes of Health (NIH) Guide for the Care and Use of Laboratory Animals. Additionally, 6- to 8-week-old female WT BALB/c mice received FMT from 7-week-old healthy female WT or MIF-KO donors bred and housed in our vivarium. Throughout the experiments, all the mice were housed in pathogen-free environments with ad libitum access to water and food (Laboratory Rodent Diet 5001, Cat. #3005659-220; LabDiet, Richmond, IN, USA).

### 2.2. Fecal Microbiota Transplantation

**Figure 1:**
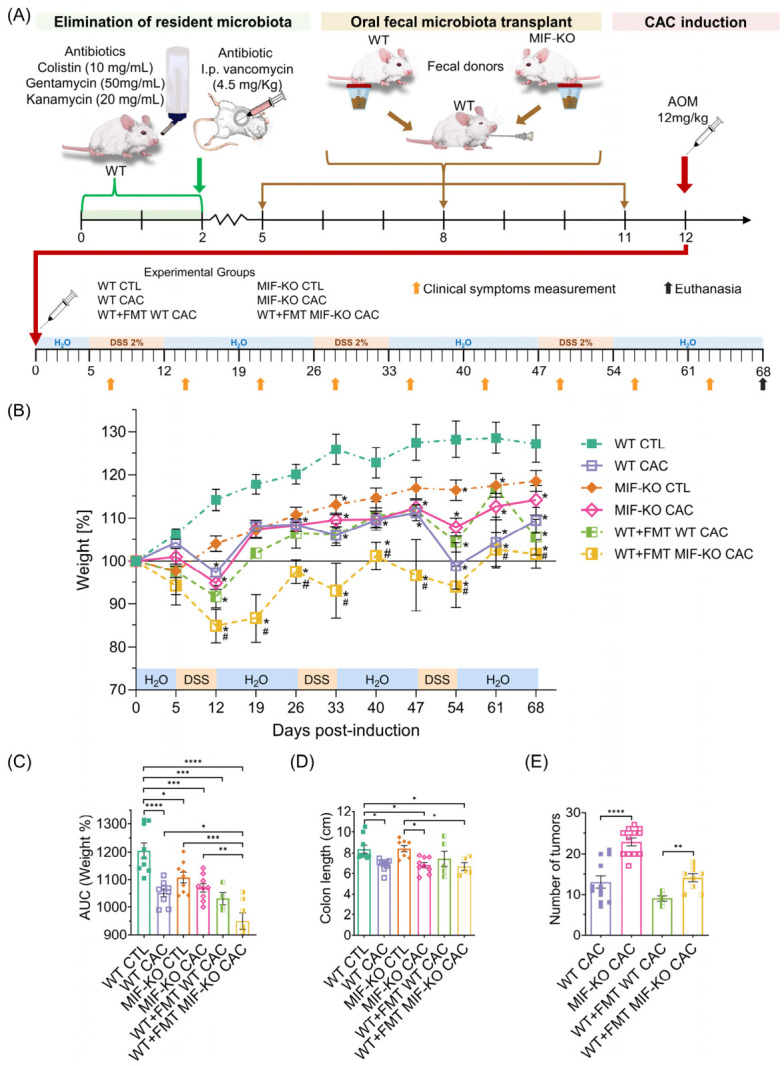
The absence of MIF enhances CAC development. Body weight was monitored for 68 days during AOM/DSS-induced CAC. (**A**) Experimental timeline: WT mice were antibiotic-treated to deplete the microbiota, followed by oral FMT from WT or MIF-KO donors, after which all groups received AOM/DSS. (**B**) Body weight (%); (**C**) area under the curve (AUC) of weight % in the model; (**D**) colon length (cecum–anus); and (**E**) tumor burden. AUC values represent mean individual data; n = 9. Statistical analyses: two-way ANOVA for (**A**); one-way ANOVA with Tukey’s test for (**B**–**D**); Student’s *t*-test for tumor counts comparing WT CAC vs. MIF-KO CAC and WT+FMT WT CAC vs. WT+FMT MIF-KO CAC. Analyses were performed using GraphPad Prism 8.3. * *p* < 0.05, ** *p* < 0.01, *** *p* < 0.001, **** *p* < 0.0001 vs. WT CTLs and # *p* < 0.05 vs. MIF-KO CTLs.

BALB/c WT mice were treated with an antibiotic cocktail consisting of colistin (10 mg/mL), gentamicin (50 mg/mL), and kanamycin (20 mg/mL) administered ad libitum in the drinking water (Sigma-Aldrich, St. Louis, MO, USA). Two days later, vancomycin (4.5 mg/kg) was delivered intraperitoneally in sterile saline. After a 5-day recovery period, pathogen-free WT mice received FMT from WT or MIF-KO donors (WT+FMT WT and WT+FMT MIF-KO, respectively). The FMT donors were age- (8–10-week-old) and sex-matched (female mice) to the recipients. FMT material was prepared from freshly collected fecal pellets mechanically resuspended in sterile drinking water (25 mg/100 μL). The suspension was allowed to sediment, and the supernatant was immediately recovered for administration. The FMT material was not prepared under anaerobic conditions; thus, obligate anaerobes may not have been preserved. The mice received FMT every 3 days via oral gavage using an 18G × 1.5 cannula (Cadence Science, Staunton, VA, USA) (Figure 1A).

### 2.3. Colitis-Associated Colorectal Cancer Model

CAC was induced in WT and MIF-KO mice harboring native microbiota (WT CAC and MIF-KO CAC, respectively). CAC was also induced in WT mice previously transplanted with WT and MIF-KO microbiota (WT+FMT WT CAC and WT+FMT MIF-KO CAC, respectively). All groups underwent CAC induction following the protocol described by Neufert et al. (2007) [18]. On day 0, the mice received a single intraperitoneal injection of 12 mg/kg azoxymethane (AOM) (Sigma-Aldrich), followed by treatment with dextran sulfate sodium (DSS) (Alfa Aesar, Haverhill, MA, USA). The mice then received three cycles of 2% DSS in the drinking water for 7 days, interspersed with a 14-day recovery period with regular water, starting two weeks after AOM administration. Weight loss was recorded weekly. The mice were euthanized via CO_2_ inhalation 68 days after AOM administration. The colon was dissected, and its length (from the distal cecum to the anus) was measured using a digital caliper (Mitutoyo, Kanagawa, Japan). The colons were flushed with saline and opened longitudinally to assess tumor development. The total tumor burden per mouse was defined as the sum of all individual tumors detected via macroscopic inspection of the colon. Although tumor evaluation followed the same criteria across groups, formal blinding during tumor counting was not implemented. Phenotypic analyses were performed using a full experimental cohort of n = 9.

### 2.4. 16S rRNA Sequencing

For 16S rRNA gene sequencing of the microbiota from WT and MIF-KO mice, with or without CAC, freshly collected fecal pellets were obtained over at least 3 consecutive days and stored at −70 °C until processing. Fecal samples were similarly collected from WT mice receiving FMT from in-house WT or MIF-KO donors. 16S rRNA sequencing was conducted on a subset of animals for which fecal samples were available and met the requirements for sequencing (WT and MIF-KO CTL n = 2 and WT CAC, MIF-KO CAC, WT+FMT WT CAC and WT+FMT MIF-KO CAC n = 3). Genomic DNA was extracted from each pellet using the DNeasy Blood and Tissue Kit. Samples were treated with RNase (Thermo Fisher Scientific, Waltham, MA, USA) and purified using the NucleoSpin Gel and PCR Clean-up Kit (Macherey-Nagel, Düren, Germany). DNA integrity was verified by 0.8% agarose gel electrophoresis, and the DNA concentration was quantified using a Qubit fluorometer (Thermo Fisher Scientific) with a Qubit dsDNA HS Assay Kit. The samples were concentrated using a miVac centrifugal concentrator (Genevac, Ipswich, Suffolk, UK) and adjusted to 5 ng/μL.

The V3–V4 hypervariable region of the bacterial 16S rRNA gene was amplified by PCR using the following primers: F: 5’-TCGTCGGCAGCGTCAGATGTGTATAAGAGACAGCCTACGGGNGGCWGCAG-3’ and R: 5’-GTCTCGTGGGCTCGGAGATGTGTATAAGAGACAGGACTACHVGGGTATCTAATCC-3’. Amplification was performed with GoTaq Green Master Mix (Promega, Madison, WI, USA). No-template controls were included to detect potential contamination.

PCR products were subsequently purified with Agencourt AMPure XP beads (Beckman Coulter Inc., Brea, CA, USA) using a DynaMag-96 Side Magnet (Thermo Fisher Scientific). Purified amplicons were indexed using the Nextera XT DNA Library Prep Kit (Illumina, San Diego, CA, USA) with Index 1 (N7XX) and Index 2 (S5XX) adapters and KAPA HiFi HotStart ReadyMix (Roche, Basel, Switzerland). Indexed libraries were further purified with AMPure XP beads and ethanol (Sigma-Aldrich). Sequencing was performed on the Illumina MiSeq platform (Illumina, Inc., San Diego, CA, USA).

### 2.5. Bioinformatics and Sequence Processing

V3–V4 paired-end reads of the 16S rRNA gene underwent quality control, filtering, trimming, and merging to infer amplicon sequence variants (ASVs). Processing parameters were defined on the basis of quality profile inspection and primer position verification. Forward and reverse reads were truncated to 270 and 210 nucleotides, respectively, with an initial 25-nucleotide trim applied in both directions. Reads containing ambiguous bases were excluded, and maximum expected error thresholds of two (forward) and three (reverse) were applied. Finally, chimeric sequences were removed.

### 2.6. Alpha Diversity Analysis

Following sequence processing and ASV table construction, alpha diversity was evaluated to estimate within-sample richness and diversity. The samples were rarefied to 8797 reads per sample, corresponding to the minimum retained sequencing depth across samples, before the alpha diversity indices were calculated. Rarefaction curves were generated to evaluate sequencing depth for richness estimation and are provided in Supplementary Figure S1. The observed ASVs and Chao1 index were used as richness estimators, while the Shannon and Simpson indices were calculated to assess both richness and evenness. Global differences among experimental groups were evaluated using the Kruskal–Wallis test; significant outcomes were followed by Dunn’s post hoc test for pairwise comparisons. *p*-values were adjusted for multiple testing using the false discovery rate method, and diversity patterns were visualized using boxplots.

### 2.7. Beta Diversity Analysis

Beta diversity was evaluated to estimate differences in the microbial community structure among the samples. Bray–Curtis and weighted UniFrac distances were used to assess community dissimilarities on the basis of taxon abundance alone or abundance weighted by phylogenetic relationships, respectively.

Global differences among experimental groups were assessed via permutational multivariate analysis of variance (PERMANOVA) using 999 permutations, and pairwise comparisons were performed for a priori defined, biologically relevant contrasts. *p*-values were adjusted for multiple testing using the false discovery rate method. The pairwise results were summarized using the R^2^ value, the adjusted *p*-value, and the mean between-group distance. The multivariate homogeneity of group dispersions was also evaluated with betadisper using 999 permutations to determine the influence of within-group variability. Complete pairwise beta diversity results, including R^2^ values, mean distances, adjusted *p*-values and betadisper *p*-values, are provided in Supplementary Table S1. Beta diversity patterns were visualized using principal coordinate analysis (PCoA).

### 2.8. Differential Abundance Analysis

Taxonomic differential abundance was evaluated by analyzing the compositions of the microbiomes with bias correction 2 (ANCOM-BC2) at the family and genus levels. Pairwise comparisons were performed between selected experimental groups to assess genotype-associated differences, CAC-related shifts within each genetic background, and contrasts involving FMT recipients. *p*-values were adjusted for multiple comparisons using the Benjamini–Hochberg method, and the resulting adjusted *q*-values served as the primary interpretation criteria. Comparisons for which *q* < 0.05 were considered statistically significant, whereas values between 0.05 and 0.10 were considered nonsignificant exploratory tendencies. The W statistic was retained as a complementary ANCOM-BC2 output.

Since genus-level analyses may mask variation among individual amplicon sequence variants, an additional exploratory ANCOM-BC2 analysis was performed at the ASV level without taxonomic agglomeration. Species-level assignments were not used for biological interpretation because the resolution of the V3–V4 16S rRNA region at the species level is limited and varies across bacterial taxa. The direction of each log-fold change (LFC) was aligned with the observed relative abundance of the compared groups, where positive values indicated higher abundance in the second group of the comparison and negative values indicated enrichment in the first group.

### 2.9. Statistical Analysis

Differences between groups were evaluated using one-way ANOVA, two-way ANOVA, or Student’s *t* test, as appropriate. Statistical significance was defined as *p* < 0.05 (*), *p* < 0.01 (**), *p* < 0.001 (***), or *p* < 0.0001 (****). These analyses were performed using GraphPad Prism 8.3 (GraphPad Software, Inc., San Diego, CA, USA). Bioinformatic and diversity analyses were performed using R version 4.5.2 software.

Exploratory Spearman rank correlation analyses were performed between tumor burden and the relative abundance of selected family- and genus-level taxa. Taxa were selected a priori on the basis of their representation in the relative abundance profiles and ANCOM-BC2 results. Correlations were evaluated in three predefined sample sets: all sequenced mice (WT CTL, WT CAC, MIF-KO CTL, MIF-KO CAC, WT+FMT WT CAC, and WT+FMT MIF-KO CAC), CAC-bearing mice only (WT CAC, MIF-KO CAC, WT+FMT WT CAC, and WT+FMT MIF-KO CAC), and FMT-recipient CAC mice only (WT+FMT WT CAC and WT+FMT MIF-KO CAC). *p*-values were adjusted within each analysis set and taxonomic level using the Benjamini–Hochberg method; *q* < 0.05 (*) and *q* < 0.10 (^+^). The correlation results are provided in Supplementary Table S2.

## 3. Results

### 3.1. MIF Deficiency-Associated Microbiota Exerts a Protumorigenic Effect on AOM/DSS-Induced Colitis-Associated Colorectal Cancer

MIF-KO and WT mice were subjected to the AOM/DSS protocol (Figure 1A). Although both groups developed CAC, the MIF-KO mice exhibited earlier and more severe clinical symptoms. While WT CAC mice manifested moderate symptoms starting from the second DSS cycle, MIF-KO CAC mice displayed piloerection as early as the first cycle and bloody diarrhea during the second cycle, as previously reported in our model [19].

Body weight monitoring revealed distinct patterns; both the WT CAC and MIF-KO CAC groups lost weight at the end of the DSS cycles compared with their respective controls (Figure 1B). Area under the curve (AUC) analysis revealed a significant reduction in WT CAC mice versus WT CTL mice (**** *p* < 0.0001), whereas compared with control mice, MIF-KO CAC mice did not significantly differ (Figure 1C). Nevertheless, the tumor phenotype was more severe in the absence of MIF; although both the WT CAC and MIF-KO CAC groups exhibited no significant difference in terms of colon shortening (Figure 1D), and compared with WT CAC mice (13.0 ± 1.46), MIF-KO CAC mice developed significantly more tumors (23.9 ± 1.33) (**** *p* < 0.0001) (Figure 1E).

To evaluate whether the MIF-KO microbiota contributes to tumor progression, FMT was performed. Antibiotic-pretreated WT mice received microbiota from either WT (WT+FMT WT) or MIF-KO (WT+FMT MIF-KO CAC) donors (Figure 1A).

Following CAC induction, both FMT-recipient groups showed significant weight loss compared to the WT CTL group on days 12, 33, 54, and 68 (*** *p* < 0.001). However, compared with the mice in the other experimental groups, the mice in the WT+FMT MIF-KO CAC group exhibited the most pronounced reduction in body weight after the first DSS cycle and failed to recover. This trend persisted throughout the experiment (Figure 1B), as corroborated by the results of the AUC analysis, which revealed significant differences compared with those of WT CTLs and MIF-KO CTLs, indicating that the MIF-KO microbiota severely affected weight loss, which is a hallmark of CAC development in our model (Figure 1C).

Colon length on day 68 postinduction was significantly reduced in WT+FMT MIF-KO mice (* *p* < 0.05) (Figure 1D). Assessment of tumor burden further demonstrated that the MIF-KO microbiota exacerbates carcinogenesis. WT+FMT MIF-KO CAC mice had significantly greater tumor counts than WT+FMT WT CAC mice did (* *p* < 0.05; Figure 1E).

Taken together, these data confirm that compared with WT mice, MIF-KO mice develop greater tumor burdens, which is consistent with previously reported findings [19]. Importantly, these results demonstrate that the microbiota associated with MIF deficiency exerts a transmissible protumorigenic effect.

### 3.2. Characterization of the Microbiota in WT and MIF-KO Mice with Colitis-Associated Colorectal Cancer

To determine the influence of MIF on the composition of the intestinal microbiota, 16S rRNA gene sequencing was performed on fecal samples from WT and MIF-KO mice, both under basal conditions and following CAC induction, subsequent FMT, and CAC induction. Phenotypic analyses were performed using the full experimental cohort. In contrast, 16S rRNA sequencing was conducted on a subset of animals for which fecal samples were available and met the requirements for sequencing. Therefore, microbiome analyses should be interpreted as exploratory and hypothesis-generating.

#### 3.2.1. Quality Control and Bioinformatic Processing of Sequences

The final pipeline yielded an overall chimera rate of 3.57% and a median of 14,738 nonchimeric reads per sample (range: 8797–17,868). Taxonomic assignment was carried out against the SILVA NR99 v138.2 database; subsequent downstream analyses were restricted to the genus level (Table 1).

Downstream taxonomic summaries were performed mainly at the phylum, family, and genus levels, and an additional exploratory ASV-level differential-abundance analysis was performed as described below.

#### 3.2.2. Alpha Diversity Analysis Revealed Global Variation Without Significant Selected Pairwise Differences

**Figure 2:**
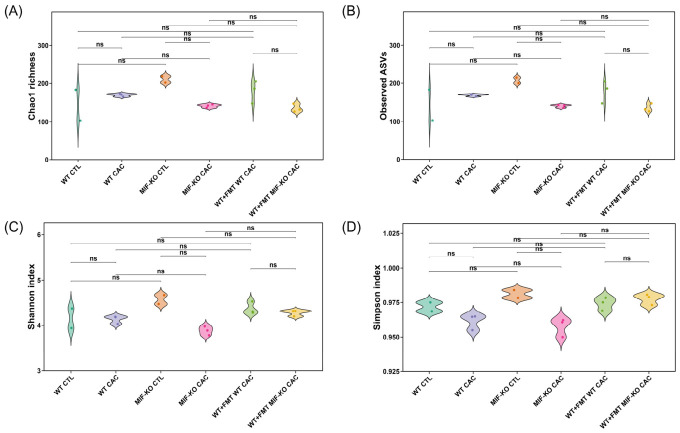
Microbial alpha-diversity in stool samples from the WT CTL, WT CAC, MIF-KO CTL, MIF-KO CAC, WT+FMT WT CAC, and WT+FMT MIF-KO CAC groups. (A) Chao1 richness; (B) observed ASV richness; (C) Shannon diversity; and (D) Simpson diversity. Global differences were evaluated using the Kruskal–Wallis test, and selected pairwise comparisons were assessed using Dunn’s post hoc test with false discovery rate correction. Although the global Kruskal–Wallis values of the Shannon and Simpson indices were significant, no selected pairwise comparisons remained significant after correction. WT and MIF-KO CTLs; n = 2; and WT CAC, MIF-KO CAC, WT+FMT WT CAC and WT+FMT MIF-KO CAC; n = 3. ns = not significant.

The richness indices Chao1 and observed ASVs, in addition to the Shannon and Simpson diversity indices, were used to describe the alpha diversity of the colonic microbiota across the experimental groups. These indices were calculated after all the samples were rarefied to 8797 reads per sample. Rarefaction curves revealed that most samples approached a plateau near this sequencing depth, supporting its use for alpha diversity estimation (Supplementary Figure S1). The results of the Kruskal–Wallis test revealed global differences in the Shannon (* *p* = 0.0491) and Simpson (*p = 0.0298) indices, whereas the differences in the Chao1 (*p* = 0.0671) and observed ASVs (*p* = 0.0757) indices did not reach statistical significance. However, after correction for multiple testing, no statistically significant differences were detected in the selected pairwise comparisons. Thus, the global alpha diversity variation did not translate into statistically supported selected pairwise differences. Given the limited number of sequenced samples, these nonsignificant pairwise results should not be interpreted as definitive evidence of the absence of biological differences among groups (Figure 2; Table 2, Appendix A).

Together with the taxonomic profiles, these results suggest that the observed protumorigenic phenotype was accompanied by selective changes in microbial composition rather than by a statistically supported pairwise loss of overall alpha diversity.

#### 3.2.3. MIF Deficiency Modulates CAC-Associated Microbial Community Convergence

Principal coordinate analyses (PCoAs) based on Bray–Curtis and weighted UniFrac distance metrics were performed to determine variations in the composition and phylogenetic structure of the microbial community across the different experimental groups. Global PERMANOVA indicated significant overall differences among groups for both beta diversity metrics using 999 permutations (*** *p* = 0.001). However, none of the selected pairwise PERMANOVA comparisons remained statistically significant after Benjamini–Hochberg correction. Therefore, the pairwise beta diversity results were interpreted as exploratory effect size patterns rather than as statistically significant group separations. The complete pairwise R^2^ values, mean distances, adjusted *p*-values, and betadisper *p*-values are shown in Supplementary Table S1.

**Figure 3:**
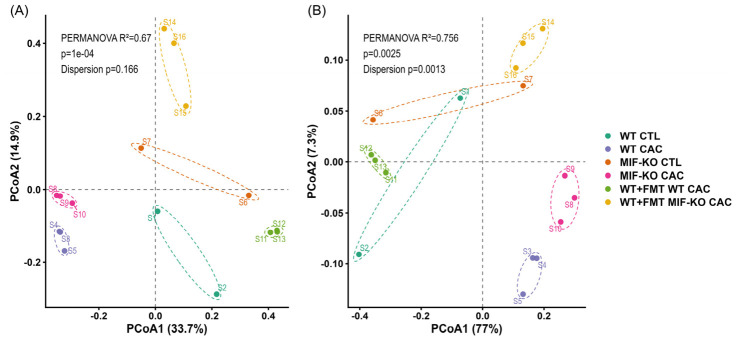
Microbial beta-diversity in stool samples from the WT CTL, WT CAC, MIF-KO CTL, MIF-KO CAC, WT+FMT WT CAC and WT+FMT MIF-KO CAC groups. (**A**) PCoA of the Bray–Curtis distances showing separation among the healthy, CAC, and FMT groups (global PERMANOVA, using 999 permutations). (**B**) Weighted UniFrac PCoA revealing differences between the healthy control, WT vs. MIF-KO FMT recipient and the WT vs. MIF-KO CAC groups (global PERMANOVA, using 999 permutations). WT and MIF-KO CTLs; n = 2; and WT CAC, MIF-KO CAC, WT+FMT WT CAC and WT+FMT MIF-KO CAC; n = 3.

In the Bray–Curtis analysis, the most pronounced signals were observed in the comparisons of WT CAC vs. WT+FMT WT CAC (R^2^ = 0.866, adjusted *p* = 0.118; average distance = 0.822) and WT+FMT WT CAC vs. WT+FMT MIF-KO CAC (R^2^ = 0.604, adjusted *p* = 0.118; average distance = 0.758). Although these differences did not reach statistical significance, they suggest a trend toward compositional separation between tumor-bearing groups and FMT-associated groups (Figure 3A).

When weighted UniFrac was used, several biologically relevant contrasts showed higher R^2^ values than those observed with Bray–Curtis, although none of the corresponding adjusted *p*-values reached statistical significance. The greatest R^2^ values were observed for WT CAC vs. WT+FMT WT CAC (R^2^ = 0.919; adjusted *p* = 0.144; mean distance = 0.533), MIF-KO CAC vs. WT+FMT WT CAC (R^2^ = 0.919; adjusted *p* = 0.144; mean distance = 0.635), and WT+FMT WT CAC vs. WT+FMT MIF-KO CAC (R^2^ = 0.874; adjusted *p* = 0.144; mean distance = 0.527). Importantly, the comparison between both FMT-recipient groups did not show evidence of differential dispersion by betadisper analysis (*p* = 0.801), suggesting that this pattern was not driven mainly by differences in within-group variability. In contrast, several comparisons involving control groups showed significant betadisper values, indicating that these PERMANOVA results should be interpreted with additional caution. Therefore, the beta diversity results were considered exploratory and were interpreted together with the phenotypic and taxonomic findings rather than as evidence of statistically significant pairwise separation (Figure 3B; Supplementary Table S1).

#### 3.2.4. Taxa Associated with Dysbiosis and Tumor Progression

To identify the taxonomic changes responsible for the clustering patterns observed in the PCoA, relative abundances were analyzed across different taxonomic levels. At the phylum level under basal conditions, the WT CTL and MIF-KO CTL groups exhibited similar distributions of the major bacterial phyla, with the exception of the phylum Pseudomonata, whose abundance was greater in the WT CTL group.

**Figure 4:**
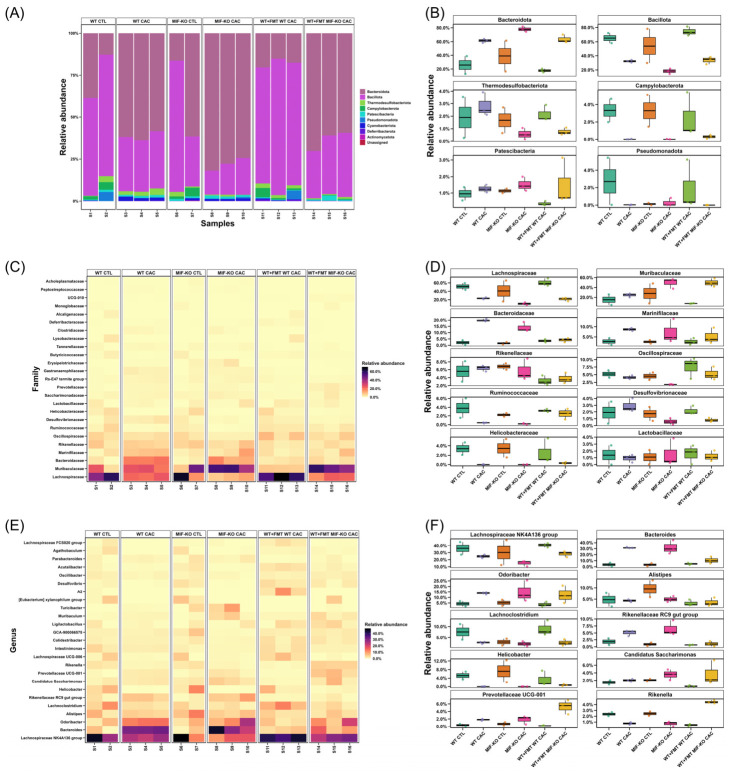
Changes in the intestinal microbiota across experimental groups. (**A**) Relative abundance of the top 10 phyla. (**B**) Boxplots showing the phyla whose abundance differed significantly among the groups. (**C**) Heatmap of the 25 most abundant bacterial families. (**D**) Boxplots of the six families showing the most significant changes. (**E**) Heatmap of the 25 most abundant genera. (**F**) Boxplot of the top six significantly altered microbial communities at the genus level. WT and MIF-KO CTLs; n = 2; and WT CAC, MIF-KO CAC, WT+FMT WT CAC and WT+FMT MIF-KO CAC; n = 3.

In both genetic backgrounds, under both CTL conditions and following CAC induction, Bacteroidota and Bacillota constituted the predominant phyla. Importantly, the development of CAC induced an increase in the proportions of Bacteroidota, Thermodesulfobacteroidota, and Patescibacteria, along with a reduction in Bacillota and Campylobacterota in the WT CAC, MIF-KO CAC, and WT+FMT MIF-KO CAC groups, thereby altering the basal equilibrium. In particular, compared with WT CAC mice, MIF-KO CAC mice displayed an even greater proportion of Bacteroidota and a more pronounced reduction in Bacillota abundance. Notably, the relative abundances of the six major phyla in the WT+FMT WT CAC group were similar to those in the WT CTL group, indicating less exacerbated dysbiosis (Figure 4A,B).

At the family level, the WT CTL and MIF-KO CTL groups again exhibited similar distribution profiles among the major bacterial families. CAC development induced a decrease in the proportions of Lachnospiraceae and Oscillospiraceae and an increase in Muribaculaceae, Bacteroidaceae, and Marinifilaceae in the WT CAC, MIF-KO CAC, and WT+FMT MIF-KO CAC groups. Specifically, the proportion of Lachnospiraceae was lower in MIF-KO CAC mice than in WT CAC mice. In the case of Rikenellaceae, only the groups subjected to both FMT and CAC tended to decrease in relative abundance; with the exception of this family, the WT+FMT WT CAC group again displayed relative abundance profiles for the six major families that closely resembled those of the WT CTL group (Figure 4C,D).

At the genus level, relevant differences in microbial composition were identified among the experimental groups. Under basal CTL conditions, both WT and MIF-KO mice presented a high abundance of the *Lachnospiraceae NK4A136* group. In contrast, the abundance of the genus *Alistipes* was greater in MIF-KO CTL mice than in WT CTL mice. Following CAC induction, the abundances of the *Lachnospiraceae NK4A136* group, *Alistipes*, and *Lachnoclostridium* decreased in WT CAC and MIF-KO CAC, whereas the relative abundances of *Bacteroides, Odoribacter*, and the *Rikenellaceae RC9* gut group increased. Notably, MIF-KO CAC mice presented a more pronounced reduction in the *Lachnospiraceae NK4A136* group compared to WT CAC.

In the groups that received FMT and subsequently had CAC, the abundances of genera such as *Alistipes* and *Rikenellaceae RC9* gut group were reduced in both FMT groups compared with those in the nontransplanted CAC groups. Notably, the relative abundance of the six major genera was maintained at values similar to those of WT CTL in WT+FMT WT CAC, demonstrating greater resilience to the dysbiosis observed after the AOM/DSS protocol. Conversely, the abundance of Bacteroides was significantly greater in the WT+FMT MIF-KO CAC group than in the WT+FMT WT CAC group (Figure 4E,F).

Collectively, the relative abundance of the six major genera present in WT+FMT WT CAC suggests less severe dysbiosis, remaining similar to that found in WT CTL mice. In contrast, WT+FMT MIF-KO CAC exhibited greater variation, with a profile closer to that of MIF-KO CAC and, therefore, severe dysbiosis.

#### 3.2.5. Taxonomic Differential Abundance Analysis via ANCOM-BC2

**Figure 5:**
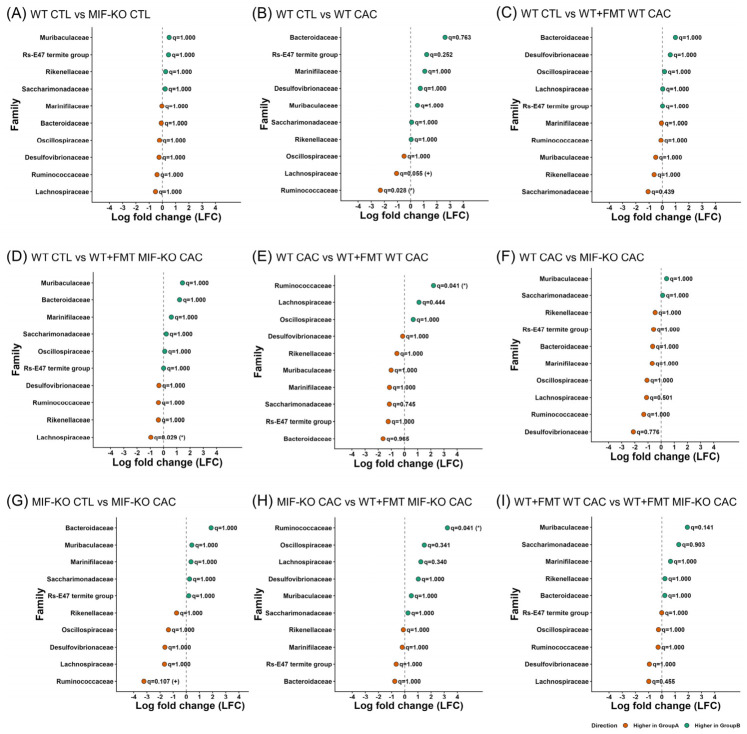
Differential abundance of dominant bacterial families across experimental groups using ANCOM-BC2. (**A**) WT CTL vs. MIF-KO CTL, (**B**) WT CTL vs. WT CAC, (**C**) WT CTL vs. WT+FMT WT CAC, (**D**) WT CTL vs. WT+FMT MIF-KO CAC, (**E**) WT CAC vs. WT+FMT WT CAC, (**F**) WT CAC vs. MIF-KO CAC, (**G**) MIF-KO CTL vs. MIF-KO CAC, (**H**) MIF-KO CAC vs. WT+FMT MIF-KO CAC and (**I**) WT+FMT WT CAC vs. WT+FMT MIF-KO CAC. Positive log-fold change (LFC) values indicate enrichment in the second group of the contrast, whereas negative values indicate enrichment in the first group. Complete values, including the W statistic, *p*-value, *q*-value (* *q* < 0.05, ^+^ *q* < 0.10), and significance status, are provided in Supplementary Table S3. WT and MIF-KO CTLs; n = 2; and WT CAC, MIF-KO CAC, WT+FMT WT CAC and WT+FMT MIF-KO CAC; n = 3.

**Figure 6:**
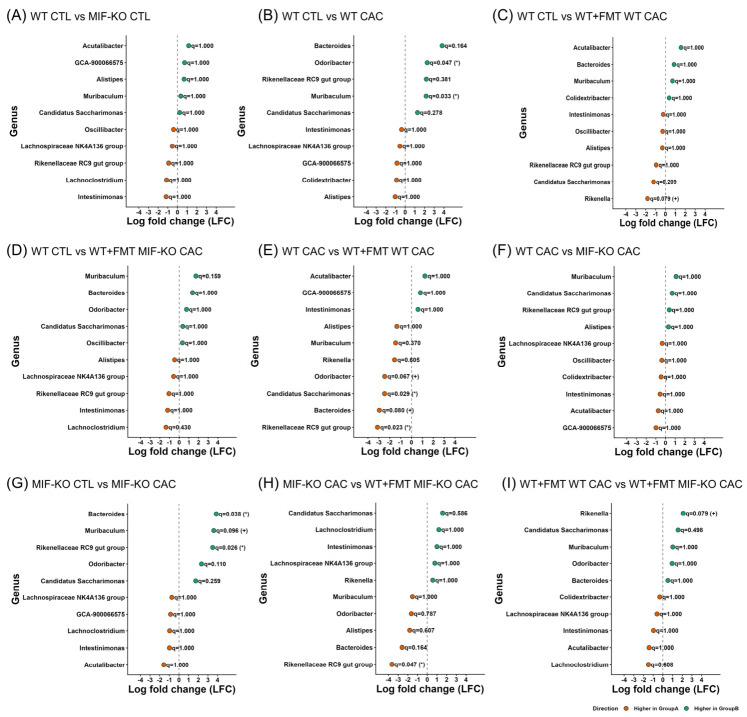
ANCOM-BC2 differential abundance of the top genera between the experimental groups. (**A**) WT CTL vs. MIF-KO CTL, (**B**) WT CTL vs. WT CAC, (**C**) WT CTL vs. WT+FMT WT CAC, (**D**) WT CTL vs. WT+FMT MIF-KO CAC, (**E**) WT CAC vs. WT+FMT WT CAC, (**F**) WT CAC vs. MIF-KO CAC, (**G**) MIF-KO CTL vs. MIF-KO CAC, (**H**) MIF-KO CAC vs. WT+FMT MIF-KO CAC and (**I**) WT+FMT WT CAC vs. WT+FMT MIF-KO CAC. Positive log-fold change (LFC) values indicate enrichment in the second group of the contrast, whereas negative values indicate enrichment in the first group. Complete values, including the W statistic, *p*-value, *q*-value (* *q* < 0.05, ^+^ *q* <0 1), and significance status, are provided in Supplementary Table S3. WT and MIF-KO CTLs; n = 2; and WT CAC, MIF-KO CAC, WT+FMT WT CAC and WT+FMT MIF-KO CAC; n = 3.

Differential abundance was evaluated using ANCOM-BC2 at the family and genus levels. The * *q* < 0.05 threshold was applied consistently to define statistically significant differences, whereas adjusted ^+^*q*-values between 0.05 and 0.10 were treated only as nonsignificant exploratory tendencies. The family- and genus-level outputs corresponding to Figure 5 and Figure 6, including the LFC, W statistic, *p*-value, *q*-value and significance status, are provided in Supplementary Table S3. A summary of the number of significant features detected in each comparison is also provided in Supplementary Table S3A.

At the family level, the comparison between WT CTL and MIF-KO CTL revealed no significant differences after multiple-testing correction (*q* = 1.000; Figure 5A), suggesting that both control groups started from globally similar profiles at this taxonomic level.

Within the WT background, the transition from WT CTL to WT CAC showed discrete yet detectable changes. The abundance of Ruminococcaceae decreased significantly (*q = 0.028), whereas that of Lachnospiraceae did not significantly decrease after correction (^+^*q* = 0.055; Figure 5B). No families reached statistical significance in the WT CTL vs. WT+FMT WT CAC comparison after correction (Figure 5C; Supplementary Table S3A). Conversely, the comparison between WT CTL and WT+FMT MIF-KO CAC revealed a significant decrease in Lachnospiraceae abundance (* *q* = 0.029; Figure 5D). Furthermore, the contrast between WT CAC and WT+FMT WT CAC indicated a significant increase in Ruminococcaceae (* *q* = 0.041; Figure 5E). Direct comparisons between WT CAC and MIF-KO CAC did not reveal significant family-level features after correction (Figure 5F; Supplementary Table S3A). Therefore, ANCOM-BC2 did not significantly support family-level differences between these CAC groups after correction, but rather demonstrated that their microbial communities were identical.

When the MIF-KO CTL and MIF-KO CAC groups were compared, Ruminococcaceae showed a non-significant decrease after correction (Figure 5G). These findings indicate that CAC induction was associated with selected family-level shifts, although the number of statistically significant features was limited.

In contrast, Ruminococcaceae was significantly more abundant in WT+FMT WT CAC recipients than in their WT CAC donors (* *q* = 0.041). Similarly, compared with their MIF-KO CAC donors, WT+FMT MIF-KO CAC recipients presented a significantly greater abundance of Ruminococcaceae (* *q* = 0.041; Figure 5H).

Finally, in the comparison between the two FMT recipient groups (WT+FMT WT CAC vs. WT+FMT MIF-KO CAC), none of the highlighted families reached statistical significance after correction (Figure 5I). Accordingly, no statistically significant family-level features were detected between the recipient communities after correction, despite their marked differences in tumor numbers.

At the genus level, the comparison between WT CTL and MIF-KO CTL revealed no significant differences (*q* = 1.000; Figure 6A), suggesting that both control groups started from globally similar profiles at this taxonomic level. In the WT background, CAC induction was associated with a limited yet defined differential alteration. The abundance of *Muribaculum* was greater in the WT CAC than in the WT CTL (* *q* = 0.033), whereas the abundance of *Odoribacter* was also greater in the CAC-bearing group (* *q* = 0.047). The *Rikenellaceae RC9* gut group shifted in the same direction, although statistical support was lacking after multiple-testing correction (^+^ *q* = 0.381; Figure 6B).

These results suggest that progression toward CAC in WT mice is accompanied by detectable genus-level dysbiosis. In MIF-deficient animals, a partially similar but nonidentical pattern was observed. When WT CTL was compared with WT+FMT WT CAC, *Rikenella* showed a non-significant exploratory decrease (^+^ *q* = 0.079; Figure 6C). Conversely, in the comparison between WT CTL and WT+FMT MIF-KO CAC, no genus reached statistical significance after correction (Figure 6D; Supplementary Table S3A). When WT CAC was compared with WT+FMT WT CAC, the abundance of *the Rikenellaceae RC9* gut group (* *q* = 0.023) and *Candidatus Saccharimonas* (* *q* = 0.029) significantly increased, whereas the abundance of *Bacteroides* (^+^ *q* = 0.080) and *Odoribacter* (^+^ *q* = 0.067) did not significantly increase in the same direction (Figure 6E).

The contrast between WT CAC and MIF-KO CAC (Figure 6F) revealed no significant differences (*q* = 1.000) among the highlighted genera, despite their phenotypic differences in tumor burden. This result should not be interpreted as evidence that the tumor-associated communities were identical. Rather, it may reflect that CAC induction produced partially shared genus-level shifts in both genotypes, whereas differences related to tumor burden may involve lower taxonomic levels, microbial functions, or host-dependent immune conditions that are not fully resolved by genus-level 16S rRNA profiling.

In the comparison between MIF-KO CTL and MIF-KO CAC, *Bacteroides* (* *q* = 0.038) and the *Rikenellaceae RC9* gut group (* *q* = 0.026) were enriched in the tumor state, whereas *Muribaculum* showed a nonsignificant increase in the same direction (^+^ *q* = 0.096; Figure 6G). Collectively, these data indicate that CAC induced dysbiosis in both genotypes but with a differential signature depending on the host context. In the case of the WT+FMT MIF-KO CAC group, the abundance of *Rikenellaceae RC9* in the gut decreased significantly compared with that in the MIF-KO CAC group (* *q* = 0.047; Figure 6H).

Finally, comparisons between the recipient groups (WT+FMT WT CAC vs. WT+FMT MIF-KO CAC) revealed limited segregation. *Rikenella* showed a nonsignificant exploratory signal toward higher abundance in recipients that received the MIF-KO microbiota (^+^ *q* = 0.079), whereas the remaining genera showed no adjusted statistical support (Figure 6I). Therefore, although both recipient groups differed in their tumor phenotypes, this difference was not reflected in statistically supported genus-level separation.

To evaluate whether genus-level comparisons could mask subtler changes among individual sequence variants, we performed an additional exploratory ASV-level ANCOM-BC2 analysis. This analysis revealed a limited number of significant ASVs in the selected comparisons, mainly involving WT CTL vs. WT CAC and comparisons with WT recipients of MIF-KO microbiota (Supplementary Table S4). However, no significant ASV-level differences were detected between direct WT CAC and MIF-KO CAC or between WT+FMT WT CAC and WT+FMT MIF-KO CAC after multiple-testing correction. These results support a conservative interpretation of the genus-level findings and indicate that finer taxonomic or functional variation would require larger sequencing cohorts and metagenomic or strain-resolved approaches.

To further explore the relationships between the selected taxa and tumor outcome, we performed exploratory correlations between tumor number and relative abundance (Supplementary Table S2). Across all sequenced mice, tumor burden correlated positively with the families Bacteroidaceae (* *q* = 0.023) and Muribaculaceae (* *q* = 0.041) and with the genera *Muribaculum* (* *q* = 0.018) and *Bacteroides* (* *q* = 0.034). Conversely, tumor burden correlated negatively with the families Ruminococcaceae (* *q* = 0.023), Lachnospiraceae (* *q* = 0.023), and Oscillospiraceae (* *q* = 0.044), as well as with the genus-level *Lachnospiraceae NK4A136* group (* *q* = 0.048). When the analysis was restricted to CAC-bearing mice, negative associations remained significant for the families Oscillospiraceae (* *q* = 0.022), Lachnospiraceae (* *q* = 0.031), and Ruminococcaceae (* *q* = 0.039) and for the genus-level *Lachnospiraceae NK4A136* group (* *q* = 0.014), while the genus *Candidatus Saccharimonas* showed a positive association (* *q* = 0.014). In FMT-recipient mice, no correlation remained significant after correction, and this subset was therefore interpreted as exploratory.

## 4. Discussion

Accumulating evidence in IBD and colorectal cancer indicates that inflammation and the gut microbiota engage in a dynamic bidirectional interaction that reshapes the intestinal ecosystem and influences tumor progression through barrier dysfunction and amplification of immune signaling [4]. Within this framework, MIF has generally been regarded as a proinflammatory mediator that promotes tumor-associated processes, although its biological effects in colorectal cancer appear to depend on the experimental context [21,22,23,24]. Our findings add an important layer to this view by showing that, in CAC, MIF also acts as a host factor modulating the microbiota–tumor axis under the conditions of this study.

The most immediate biological observation revealed that MIF deficiency was associated with earlier clinical manifestations and a greater tumor burden in the AOM/DSS model. Importantly, this phenotype was at least partially transferable through FMT, since WT recipients of MIF-KO-derived microbiota developed a more severe tumor phenotype than did recipients of WT microbiota. However, the results of the FMT experiments should be interpreted in light of several methodological limitations. First, broad-spectrum antibiotic pretreatment may independently influence host immunity, epithelial barrier function, microbial load and tumor susceptibility [25,26,27]. Although this approach is consistent with established experimental methods in the field and has been widely used to reduce or remodel the native microbiota before FMT or microbial gavage in murine models of colorectal cancer and colitis-associated carcinogenesis [28,29], antibiotics are not biologically neutral. Therefore, our FMT findings should be interpreted with caution and in the context of potential antibiotic-related effects.

Second, potential cage effects and donor variability cannot be excluded. Therefore, the FMT findings should be considered supportive of an association between the donor microbiota source and tumor-related outcomes under the experimental conditions used rather than definitive evidence of direct microbial transmission of a tumor-promoting phenotype. Third, the interpretation of the FMT experiments is limited by the absence of additional control groups. In particular, WT recipients of WT or MIF-KO microbiota without CAC induction were not included; hence, we cannot determine whether MIF-KO-associated microbiota alone is sufficient to induce pathological changes in the absence of AOM/DSS-induced inflammation. In addition, although post-FMT microbiota profiling revealed community-level remodeling in recipient mice, donor-associated microbial differences were not longitudinally tracked throughout the experiment, and direct engraftment efficiency was not assessed. Finally, transient inflammatory responses associated with FMT itself may have contributed to tumor susceptibility. Future studies including non-CAC FMT controls, longitudinal donor–recipient microbiota analyses, and antibiotic/FMT control groups will be needed to clarify the specific contribution of MIF-KO-associated microbiota to CAC-related outcomes.

Another limitation is that formal blinding during tumor counting was not implemented, which may introduce potential observer bias despite the use of standardized macroscopic criteria. Additionally, only female mice were included; therefore, potential sex-dependent effects on the MIF–microbiota–CAC axis cannot be excluded. Nevertheless, some of the microbial alterations observed here are consistent with those reported by Liu et al. [28] in a male C57BL/6 mouse model of colorectal cancer development, in which gut microbiota profiling also revealed disease-associated ecological remodeling, including changes in Muribaculaceae and Bacteroidaceae. Thus, although our study provides a female-based perspective, its partial agreement with findings obtained in male mice suggests that some CAC-associated microbiota shifts may be conserved across sex. Future studies directly comparing male and female mice will be necessary to define which components of the MIF-associated microbial response are sex dependent and which may represent more general features of inflammation-associated colorectal carcinogenesis.

At the community level, alpha diversity analyses revealed some global variation, but no selected pairwise differences remained significant after correction for multiple testing. Therefore, these results do not demonstrate a statistically supported pairwise loss of richness or diversity in the analyzed sequencing subset, although subtle differences cannot be excluded. Together with the taxonomic results, this pattern is consistent with selective ecological remodeling rather than with a broad collapse in microbial diversity.

The β-diversity analyses provided additional exploratory support for this interpretation, particularly when weighted UniFrac was used. Although the selected pairwise comparisons did not remain significant after correction for multiple testing, the R^2^ values, mean distances and PCoA distributions suggested that some community-level patterns were more evident when phylogenetic structure was considered than when richness metrics alone were evaluated. In this context, WT recipients of MIF-KO microbiota tended to show a configuration closer to tumor-associated profiles, whereas recipients of WT-derived microbiota showed a pattern more similar to control-like states. This observation is compatible with a greater capacity of the WT-derived microbiota to maintain a less dysbiotic configuration after CAC induction, although this interpretation should be considered exploratory.

These patterns are consistent with previous reports in the AOM/DSS model [28,30] and support the notion that tumor progression in CAC is linked less to indiscriminate dysbiosis than to the selective expansion or contraction of microbial groups with distinct ecological and metabolic capacities. In this context, reductions in Bacillota- and Ruminococcaceae-related lineages may reflect diminished short-chain fatty acid production, particularly that of butyrate, with potential consequences for epithelial barrier integrity, immune regulation and tumor control [30,31,32,33,34].

The genus *Bacteroides* is particularly relevant because its members span a wide functional spectrum, ranging from commensal organisms that support intestinal homeostasis to enterotoxigenic strains that drive IL-17-mediated inflammation, epithelial damage, and tumorigenesis [35,36]. Similarly, the enrichment of the *Rikenellaceae RC9* gut group and the variable behavior of *Odoribacter* suggest that the ecological context created by MIF deficiency may favor microbial functions linked to inflammation, mucus remodeling, and altered host–microbe signaling rather than a uniform taxonomic shift [37,38].

ANCOM-BC2 analysis further refined this interpretation by identifying selected family- and genus-level differences associated with CAC and FMT conditions. However, a direct comparison between WT CAC and MIF-KO CAC did not reveal statistically significant differences at the genus level. Rather than indicating that both tumor-associated communities were equivalent, this finding suggests that CAC may induce partially shared taxonomic remodeling in both genotypes, while the greater tumor burden observed in MIF-KO mice may depend on differences in microbial function, host immune context, or taxonomic variation not fully resolved by genus-level 16S rRNA profiling. Exploratory ASV-level analysis was consistent with this interpretation, as it did not reveal additional significant separation between WT CAC and MIF-KO CAC.

Exploratory taxon–tumor correlation analysis provided additional support for the association between CAC severity and specific microbial configurations. The positive correlations between tumor burden and Bacteroidaceae/*Bacteroides* are consistent with the findings of previous reports that identified *Bacteroides* as one of the genera altered during colorectal inflammation and cancer development in mice. Similarly, the association with Muribaculaceae/*Muribaculum* may reflect CAC-related remodeling of a highly prevalent murine gut family with broad carbohydrate-degrading potential, although its role in intestinal inflammation and tumorigenesis appears to be context-dependent [39,40]. In contrast, the negative associations observed for Lachnospiraceae, Ruminococcaceae, Oscillospiraceae, and the *Lachnospiraceae NK4A136* group are consistent with the loss of bacterial groups commonly linked to fiber fermentation and short-chain fatty acid production, particularly butyrate, which has been associated with epithelial barrier maintenance, immune regulation, and protection against intestinal inflammation and colorectal tumor progression [41]. Importantly, several of these negative associations remained significant when only CAC-bearing mice were analyzed, suggesting that they were not explained solely by the contrast between healthy and tumor-bearing animals. However, because these analyses were exploratory and based on a limited sequencing cohort, they should be interpreted as supportive associations rather than evidence of direct taxon-specific effects on tumor development. In FMT-recipient mice, no correlation remained significant after correction, which is consistent with the limited sample size of this subset and reinforces the need for larger studies with longitudinal donor–recipient microbiota tracking.

These findings are consistent with the broader abundance patterns observed in the ANCOM-BC2 analysis, particularly the CAC-associated changes detected for *Muribaculum* and *Bacteroides* at the genus level and the reduced abundance of Ruminococcaceae in WT CAC mice. Importantly, several negative associations remained significant when only CAC-bearing mice were analyzed, suggesting that these patterns were not driven exclusively by the contrast between control and tumor-bearing animals. Taken together, these findings suggest that the phenotypic, diversity, taxonomic, and differential abundance data converge on a consistent association between MIF deficiency, gut microbiota remodeling and increased CAC severity. In this model, MIF may contribute not only to classical immune regulation but also to the ecological context in which inflammation-associated colorectal carcinogenesis develops. Nevertheless, these data do not establish whether the observed microbial changes directly promote tumor susceptibility or reflect secondary remodeling driven by the inflammatory tumor microenvironment.

This integrative view also explains why the most severe tumor phenotypes were not necessarily associated with the most dramatic changes in diversity metrics. Rather, all the data support a model in which selective ecological changes, especially those affecting phylogenetically and functionally relevant taxa, have disproportionate consequences for disease outcome. In this sense, the microbiota associated with MIF deficiency appears more permissive to tumor-promoting processes and capable of conferring susceptibility to a new host.

A major limitation of this study is the small number of biological replicates included in the 16S rRNA sequencing analysis. Although the phenotypic analyses were performed using the full experimental cohort, microbiome profiling was conducted in a smaller subset of animals. This limits statistical power, increases the potential influence of interindividual variability, and reduces the robustness of differential abundance findings. For this reason, the microbial changes reported here should be interpreted as exploratory associations that require validation in larger cohorts with sequencing sample sizes matched to the results of the phenotypic analyses. To avoid overinterpretation, pairwise comparisons and ANCOM-BC2 differential-abundance results were evaluated conservatively, considering adjusted *q*-values together with effect-size patterns and their consistency with the overall taxonomic and phenotypic findings. Thus, the microbiome data were not treated as standalone evidence but rather integrated with the tumor phenotype to provide an initial view of how MIF deficiency may be associated with microbiota configurations linked to CAC severity.

Another limitation of this study is the absence of functional validation of the microbial shifts associated with MIF deficiency. Although 16S rRNA profiling revealed changes in microbiota composition, we did not perform metabolomic analyses, SCFA quantification, functional metagenomics, or pathway prediction. Therefore, the mechanistic connection between these microbial alterations and tumor progression remains indirect. Future studies integrating microbial functional profiling, metabolite measurements, and taxon-specific validation will be necessary to define the pathways through which changes in the MIF-associated microbiota may influence CAC progression.

Additionally, immune parameters were not measured in parallel with microbiome profiling in the same animals. Previous work from our group revealed that MIF deficiency in a CAC model is associated with increased IL-17 expression and reduced numbers of tumor-associated macrophages; however, the present study does not directly determine whether these immune alterations are linked to the microbial shifts observed here. Thus, it remains unclear whether MIF deficiency promotes microbiota remodeling through direct effects on mucosal immunity, antimicrobial responses, or epithelial barrier function or whether microbial changes occur secondarily as a consequence of altered inflammation during CAC progression. Accordingly, our findings should be interpreted as preliminary evidence of a potential relationship between MIF deficiency, gut microbiota composition, and CAC severity. Future studies integrating microbiome profiling with cytokine measurements, immune cell infiltration, barrier integrity analyses, and microbial functional validation in the same animals will be needed to define the directionality and mechanisms of this immune–microbiota interaction.

While this study presents certain limitations, which restrict the ability to establish direct causal relationships, it provides a valuable first association between MIF deficiency, gut microbiota remodeling and CAC severity rather than identifying a specific microbial or metabolite-mediated mechanism. The microbiome findings were interpreted in an integrated manner, considering their agreement with the tumor phenotype, beta diversity effect size patterns, and taxonomic profiles. Future studies including larger sequencing cohorts, metagenomics, metabolomics, and absolute quantification will be important to validate these observations and define the underlying functional mechanisms [42,43,44].

Despite these limitations, the consistency of the phenotypic and microbiome-associated patterns observed here, together with their agreement with previous CAC studies such as Liu et al., supports the biological robustness of our findings. Overall, our study provides evidence that MIF is a relevant host modulator of the microbiota–tumor axis in CAC and establishes a conceptual basis for future mechanistic studies on how immune mediators shape cancer-promoting microbial ecosystems. Along with studies including post-FMT longitudinal microbiota profiling, engraftment efficiency analyses, blinded tumor assessment and taxon- or metabolite-level validation will be necessary to confirm and mechanistically define these effects. In addition, both male and female mice will be necessary to determine whether the observed MIF–microbiota–CAC associations are conserved across sex.

## 5. Conclusions

In summary, our findings suggest that MIF is associated with host-dependent modulation of the microbiota–tumor axis in CAC. MIF deficiency was associated with a more severe tumor phenotype and with selective ecological remodeling of the gut microbiota rather than with a generalized loss of microbial diversity. These changes included shifts in phylogenetically and functionally relevant taxa. In the FMT model, the microbiota from MIF-KO donors was associated with increased tumor burden in WT recipients under CAC-inducing conditions, supporting a potential relationship between MIF-dependent immune context, microbiota composition, and CAC severity. However, these findings should be interpreted as associative and hypothesis-generating, as direct microbial, metabolite-mediated, or immune mechanisms were not established in the present study (Supplementary Table S5).

## Figures and Tables

**Table 1:** Final nonchimeric read counts and observed ASVs * per sample.

Experimental Group	Sample ID	Number of Reads	Observed ASVs
WT CTL	S1	15,060	183
	S2	8797	103
WT CAC	S3	15,377	171
	S4	16,798	171
	S5	16,620	166
MIF-KO CTL	S6	17,652	200
	S7	17,868	218
MIF-KO CAC	S8	14,181	138
	S9	16,313	143
	S10	14,175	143
WT+FMT WT CAC	S11	14,417	206
	S12	15,621	186
	S13	9537	147
WT+FMT MIF-KO CAC	S14	13,253	147
	S15	9441	127
	S16	9814	132

* Final sequencing depth and observed amplicon sequence variants (ASVs) after quality filtering, trimming, paired-end merging, and chimera removal. Sample identifiers were relabeled (S1–S16) according to the plotting key used in the figures. “Number of reads” refers to the total number of non-chimeric reads retained per sample, and “observed ASVs” indicates the number of ASVs with non-zero abundance in the final non-chimeric ASV table.

**Table 2:** Average alpha-diversity index value per experimental group.

Experimental Group	Observed ASV	Chao1	Shannon	Simpson
WT CTL	143.00	143.00	4.16	0.97
WT CAC	168.33	169.37	4.13	0.96
MIF-KO CTL	207.50	210.38	4.57	0.98
MIF-KO CAC	140.33	141.13	3.88	0.96
WT+FMT WT CAC	179.33	179.57	4.37	0.97
WT+FMT MIF-KO CAC	135.33	135.33	4.28	0.98

## Data Availability

The raw RNA sequencing data generated in this study will be deposited in NCBI under accession number PRJNA1475082 and will be publicly available upon publication.

## Supplementary Materials

The following supporting information can be downloaded at: https://www.mdpi.com/article/10.3390/cimb48070712/s1.

## Abbreviations

The following abbreviations are used in this manuscript:

AOM	azoxymethane
ANCOM-BC2	analysis of compositions of microbiomes with bias correction
ASV	amplicon sequence variant
AUC	area under the curve
CAC	colitis-associated colorectal cancer
CTL	control
DSS	dextran sulfate sodium
FMT	fecal microbiota transplantation
IBD	inflammatory bowel disease
MIF	macrophage migration inhibitory factor
MIF-KO	MIF-deficient BALB/c mice
OTUs	operational Taxonomic Units
PCR	polymerase chain reaction
PCoA	principal coordinate analyses
PRRs	pattern recognition receptors
TAMs	tumor-associated macrophages
TLRs	Toll-like receptors
WT	wild type BALB/c mice

## Appendix A

**Table A1:** Pairwise Dunn’s Test post hoc results for alpha diversity indices Chao, observed, Shannon and Simpson (Kruskal–Wallis).

Comparison	Index	*p*. adj	Significance
WT CTL vs. MIF-KO CTL	Chao1	0.2226	ns
	Observed	0.2350	
	Shannon	0.4120	
	Simpson	0.4650	
WT CAC vs. MIF-KO CAC	Chao1	0.3857	ns
	Observed	0.4108	
	Shannon	0.5183	
	Simpson	0.7158	
WT+FMT WT CAC vs. WT+FMT MIF-KO CAC	Chao1	0.1980	ns
	Observed	0.2350	
	Shannon	0.7970	
	Simpson	0.7132	
WT CTL vs. WT CAC	Chao1	0.6310	ns
	Observed	0.6299	
	Shannon	0.6915	
	Simpson	0.5066	
MIF-KO CTL vs. MIF-KO CAC	Chao1	0.1605	ns
	Observed	0.1590	
	Shannon	0.0534	
	Simpson	0.0757	
WT CAC vs. WT+FMT WT CAC	Chao1	0.7003	ns
	Observed	0.7347	
	Shannon	0.4120	
	Simpson	0.2582	
WT CTL vs. WT+FMT WT CAC	Chao1	0.3857	ns
	Observed	0.4108	
	Shannon	0.5539	
	Simpson	0.7132	
MIF-KO CTL vs. WT+FMT MIF-KO CAC	Chao1	0.1605	ns
	Observed	0.1590	
	Shannon	0.4716	
	Simpson	0.7590	
MIF-KO CAC vs. WT+FMT MIF-KO CAC	Chao1	0.7970	ns
	Observed	0.8300	
	Shannon	0.1485	
	Simpson	0.0757	

ns = not significant.
