# Supplementary material for: MIF Deficiency Modulates Gut Microbiota Composition and Promotes Colitis-Associated Colorectal Cancer in a Murine Model
Source: Curr Issues Mol Biol. 2026 Jul 13;48(7):712. doi: 10.3390/cimb48070712 (PMC13407805; doi:10.3390/cimb48070712)
Supplement: Supplementary file 1 [file cimb-48-00712-s001.zip › cimb-4405355-supplementary.pdf]

Supplementary Material

Supplementary Figure S1. Rarefaction curves for ASV richness estimation.

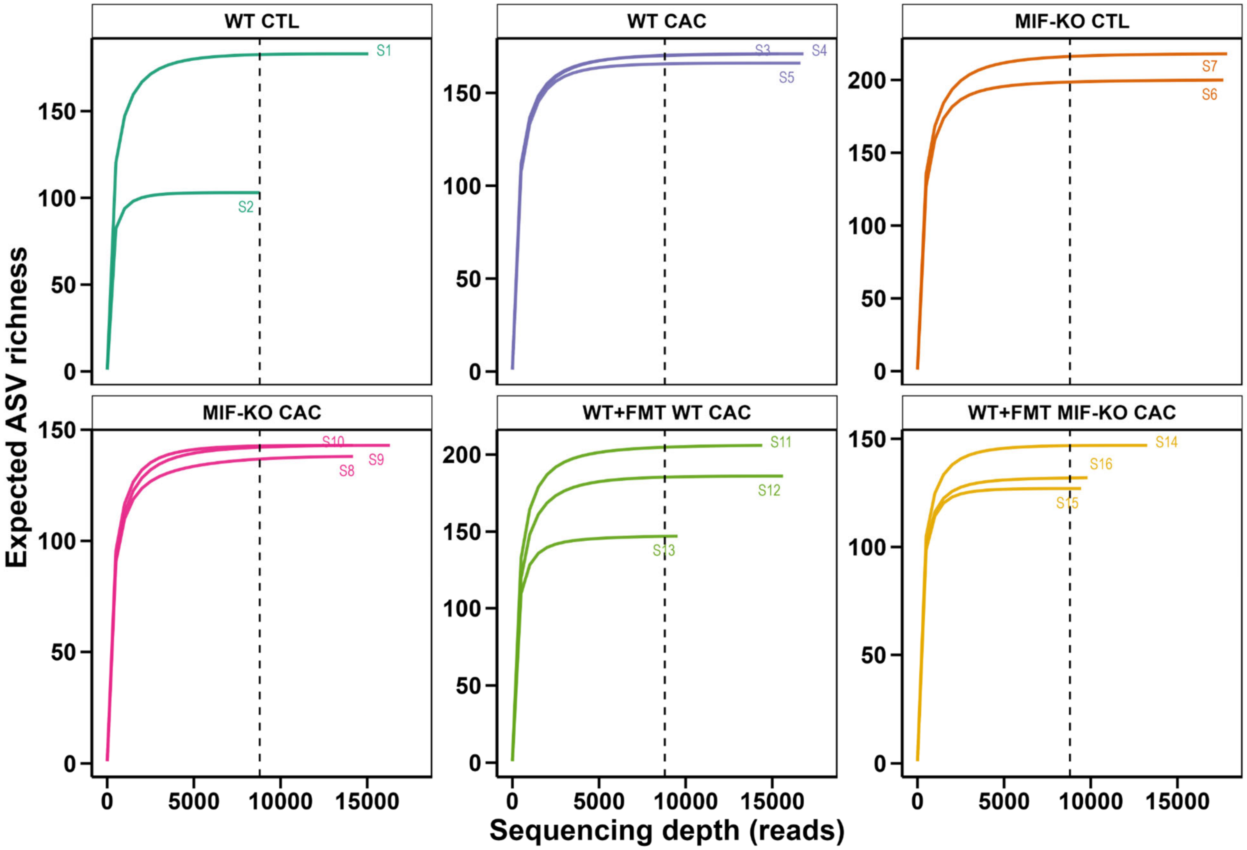

Rarefaction curves were generated for each sample to evaluate sequencing depth for richness estimation. The dashed vertical line indicates the common rarefaction depth used for alpha-diversity analysis (8,797 reads per sample), which corresponded to the minimum retained sequencing depth across samples. Most samples approached a plateau near this depth, supporting the use of a common rarefied depth for richness and alpha-diversity estimation.

Supplementary Table S1. Pairwise beta-diversity effect sizes and dispersion tests.

Mean distance represents the average between-group beta-diversity distance. Pairwise R2 indicates the proportion of variation explained by group identity in pairwise PERMANOVA. Adjusted p-values were corrected using the Benjamini–Hochberg method. Betadisper p-values were included to evaluate whether PERMANOVA patterns could be influenced by differences in within-group dispersion. This table includes the pairwise contrasts available in the beta-diversity output used for the revision.

Metric	Group A	Group B	Mean beta-diversity distance	Pairwise PERMANOVA R^2	BH-adjusted p-value	Betadisper p-value
**Bray–Curtis**	WT CTL	WT CAC	0.796	0.561	0.118	0.008
	WT CTL	MIF-KO CTL	0.803	0.300	0.667	0.042
	WT CTL	WT+FMT WT CAC	0.824	0.519	0.118	0.008
	WT CTL	WT+FMT MIF-KO CAC	0.851	0.429	0.118	0.308
	WT CAC	MIF-KO CAC	0.390	0.368	0.118	0.501
	WT CAC	WT+FMT WT CAC	0.822	0.866	0.118	0.101
	WT CAC	WT+FMT MIF-KO CAC	0.732	0.656	0.118	0.201
	MIF-KO CTL	MIF-KO CAC	0.770	0.478	0.118	0.308
	MIF-KO CTL	WT+FMT WT CAC	0.755	0.510	0.118	0.008
	MIF-KO CTL	WT+FMT MIF-KO CAC	0.729	0.345	0.325	0.908
	MIF-KO CAC	WT+FMT WT CAC	0.850	0.754	0.118	0.901
	MIF-KO CAC	WT+FMT MIF-KO CAC	0.716	0.514	0.118	0.401
	WT+FMT WT CAC	WT+FMT MIF-KO CAC	0.758	0.604	0.118	0.701
**Weighted UniFrac**	WT CTL	WT CAC	0.464	0.659	0.144	0.008
	WT CTL	MIF-KO CTL	0.364	0.097	1.000	0.042
	WT CTL	WT+FMT WT CAC	0.302	0.220	0.758	0.008
	WT CTL	WT+FMT MIF-KO CAC	0.471	0.625	0.144	0.008
	WT CAC	MIF-KO CAC	0.211	0.698	0.144	0.301
	WT CAC	WT+FMT WT CAC	0.533	0.919	0.144	0.201
	WT CAC	WT+FMT MIF-KO CAC	0.249	0.730	0.144	0.101
	MIF-KO CTL	MIF-KO CAC	0.460	0.563	0.144	0.008
	MIF-KO CTL	WT+FMT WT CAC	0.355	0.334	0.260	0.008
	MIF-KO CTL	WT+FMT MIF-KO CAC	0.359	0.361	0.355	0.008
	MIF-KO CAC	WT+FMT WT CAC	0.635	0.919	0.144	0.601
	MIF-KO CAC	WT+FMT MIF-KO CAC	0.258	0.639	0.144	0.601
	WT+FMT WT CAC	WT+FMT MIF-KO CAC	0.527	0.874	0.144	0.801

Abbreviations: BH, Benjamini–Hochberg; PERMANOVA, permutational multivariate analysis of variance; R2, coefficient of determination for the pairwise PERMANOVA model.

Supplementary Table S2. Exploratory Spearman correlations between tumor burden and selected microbiota taxa.
Spearman correlations were performed between individual tumor number and the relative abundance of selected family- and genus-level taxa. Taxa were selected a priori based on their representation in the relative-abundance profiles, ANCOM-BC2 results, and biological discussion of the manuscript. Analyses were performed across all sequenced mice, in CAC-bearing mice only, and in FMT-recipient CAC mice only. P-values were adjusted by the Benjamini–Hochberg method within each analysis set and taxonomic level.

Analysis set	Level	Taxon	n	rho	p-value	*q*-value
All sequenced mice	Family	Ruminococcaceae	16	-0.647	0.007	0.023
	Family	Lachnospiraceae	16	-0.635	0.008	0.023
	Family	Bacteroidaceae	16	0.632	0.009	0.023
	Family	Muribaculaceae	16	0.573	0.020	0.041
	Family	Oscillospiraceae	16	-0.550	0.027	0.044
	Family	Marinifilaceae	16	0.480	0.060	0.080[T]
	Family	Desulfovibrionaceae	16	-0.320	0.227	0.260
	Family	Rikenellaceae	16	-0.019	0.943	0.943
	Genus	Muribaculum	16	0.705	0.002	0.018*
	Genus	Bacteroides	16	0.632	0.009	0.034*
	Genus	Lachnospiraceae NK4A136 group	16	-0.581	0.018	0.048*
	Genus	Rikenellaceae RC9 gut group	16	0.498	0.050	0.080[N]
	Genus	Candidatus Saccharimonas	16	0.488	0.055	0.080[T]
	Genus	Odoribacter	16	0.480	0.060	0.080[T]
	Genus	Rikenella	16	-0.358	0.173	0.198
	Genus	Alistipes	16	-0.300	0.258	0.258
CAC-bearing mice only	Family	Oscillospiraceae	12	-0.780	0.003	0.022*
	Family	Lachnospiraceae	12	-0.723	0.008	0.031*
	Family	Ruminococcaceae	12	-0.681	0.015	0.039*
	Family	Muribaculaceae	12	0.603	0.038	0.075[N]
	Family	Desulfovibrionaceae	12	-0.582	0.047	0.075[N]
	Family	Bacteroidaceae	12	0.374	0.231	0.270
	Family	Rikenellaceae	12	0.370	0.236	0.270
	Family	Marinifilaceae	12	0.332	0.292	0.292
	Genus	Candidatus Saccharimonas	12	0.783	0.003	0.014*
	Genus	Lachnospiraceae NK4A136 group	12	-0.769	0.003	0.014*
	Genus	Rikenellaceae RC9 gut group	12	0.455	0.137	0.366
	Genus	Bacteroides	12	0.374	0.231	0.420
	Genus	Odoribacter	12	0.332	0.292	0.420
	Genus	Muribaculum	12	0.317	0.315	0.420
	Genus	Rikenella	12	-0.134	0.678	0.775
	Genus	Alistipes	12	0.004	0.991	0.991
FMT-recipient	Family	Desulfovibrionaceae	6	-0.841	0.036	0.288[N]

Analysis set	Level	Taxon	n	rho	p-value	*q*-value
CAC mice only	Family	Oscillospiraceae	6	-0.754	0.084	0.334[T]
	Family	Muribaculaceae	6	0.667	0.148	0.395
	Family	Lachnospiraceae	6	-0.464	0.354	0.666
	Family	Ruminococcaceae	6	-0.377	0.461	0.666
	Family	Rikenellaceae	6	0.348	0.499	0.666
	Family	Bacteroidaceae	6	-0.174	0.742	0.848
	Family	Marinifilaceae	6	-0.058	0.913	0.913
	Genus	Lachnospiraceae NK4A136 group	6	-0.754	0.084	0.395[T]
	Genus	Rikenella	6	0.696	0.125	0.395
	Genus	Candidatus Saccharimonas	6	0.667	0.148	0.395
	Genus	Alistipes	6	-0.203	0.700	0.913
	Genus	Muribaculum	6	0.203	0.700	0.913
	Genus	Bacteroides	6	-0.174	0.742	0.913
	Genus	Rikenellaceae RC9 gut group	6	-0.116	0.827	0.913
	Genus	Odoribacter	6	-0.058	0.913	0.913

rho, Spearman correlation coefficient; BH, Benjamini–Hochberg; Sig, significant after BH correction: * Significant ($q < 0.05$); [N] Nominal ($p < 0.05$ but $q \geq 0.05$); Trent [T] ($0.05 \leq p < 0.10$). Correlations were interpreted as exploratory associations and not as evidence of causality.

Supplementary Table S3. ANCOM-BC2 differential-abundance results at family and genus levels.

ANCOM-BC2 was used to evaluate differential abundance in selected biologically relevant comparisons at the family and genus levels. The W statistic, LFC, p-value, *q*-value, significance status, and the group with the higher observed mean relative abundance are provided to support the interpretation of Figures 5 and 6. Statistical significance was defined strictly as $q < 0.05$. Values with $0.05 \leq q < 0.10$ are listed only as non-significant exploratory tendencies and were not interpreted as statistically significant differences. LFC values are displayed in the direction of the observed group means: positive values indicate higher relative abundance in the second group of the contrast, whereas negative values indicate higher relative abundance in the first group.

Supplementary Table S3A. Summary of significant and exploratory family- and genus-level features by comparison.

This summary reports the number of tested features in each selected comparison and the number that met the ANCOM-BC2 adjusted significance threshold. The minimum q-value is shown to indicate the strongest adjusted signal detected in each contrast.

Taxonomic level	Comparison	Number of tested features	Significant features q < 0.05	Exploratory features 0.05 <= q < 0.10	Minimum *q*-value
Family	WT CTL vs WT+FMT WT CAC	10	0	0	0.439
	WT CAC vs WT+FMT WT CAC	10	1	0	0.041
	MIF-KO CTL vs WT+FMT WT CAC	10	0	0	1.000
	MIF-KO CAC vs WT+FMT WT CAC	10	2	0	0.026
	WT CTL vs WT+FMT MIF-KO CAC	10	1	0	0.029
	WT CAC vs WT+FMT MIF-KO CAC	10	0	1	0.092
	MIF-KO CTL vs WT+FMT MIF-KO CAC	10	0	0	1.000
	MIF-KO CAC vs WT+FMT MIF-KO CAC	10	1	0	0.041
	WT CTL vs WT CAC	10	1	1	0.028
	MIF-KO CTL vs MIF-KO CAC	10	0	0	0.107
	WT+FMT WT CAC vs WT+FMT MIF-KO CAC	10	0	0	0.141
	WT CAC vs MIF-KO CAC	10	0	0	0.501
	WT CTL vs MIF-KO CTL	10	0	0	1.000
Genus	WT CTL vs WT+FMT WT CAC	14	0	1	0.079
	WT CAC vs WT+FMT WT CAC	14	2	2	0.023
	MIF-KO CTL vs WT+FMT WT CAC	14	0	0	0.322
	MIF-KO CAC vs WT+FMT WT CAC	14	2	2	0.006
	WT CTL vs WT+FMT MIF-KO CAC	14	0	0	0.159
	WT CAC vs WT+FMT MIF-KO CAC	14	0	1	0.088
	MIF-KO CTL vs WT+FMT MIF-KO CAC	14	0	0	0.461
	MIF-KO CAC vs WT+FMT MIF-KO CAC	14	1	0	0.047
	WT CTL vs WT CAC	14	2	0	0.033
	MIF-KO CTL vs MIF-KO CAC	14	2	1	0.026

Taxonomic level	Comparison	Number of tested features	Significant features q < 0.05	Exploratory features 0.05 <= q < 0.10	Minimum *q*-value
	WT+FMT WT CAC vs WT+FMT MIF-KO CAC	14	0	1	0.079
	WT CAC vs MIF-KO CAC	14	0	0	1.000
	WT CTL vs MIF-KO CTL	14	0	0	1.000

Supplementary Table S3B. Family-level ANCOM-BC2 output including W statistics.
Detailed family-level output for the selected ANCOM-BC2 comparisons. Status indicates whether the feature was significant after Benjamini–Hochberg correction, exploratory but non-significant, or not significant.

Comparison	Taxon	LFC	W	p-value	*q*-value
WT CTL vs WT+FMT WT CAC	Saccharimonadaceae	-1.073	-2.604	0.026	0.439
	Rikenellaceae	-0.618	-0.974	1.000	1.000
	Bacteroidaceae	0.989	0.841	0.420	1.000
	Desulfovibrionaceae	0.598	0.654	0.528	1.000
	Muribaculaceae	-0.499	-0.578	0.576	1.000
	Oscillospiraceae	0.165	0.325	0.752	1.000
	Ruminococcaceae	-0.100	-0.185	0.857	1.000
	Marinifilaceae	-0.067	-0.113	0.912	1.000
	Lachnospiraceae	0.031	0.085	0.934	1.000
	Rs-E47 termite group	0.016	-0.029	0.978	1.000
WT CAC vs WT+FMT WT CAC	Ruminococcaceae	2.205	3.849	0.003	0.041*
	Lachnospiraceae	1.102	2.389	0.038	0.444
	Saccharimonadaceae	-1.150	-2.257	0.048	0.745
	Bacteroidaceae	-1.629	-2.065	0.066	0.965
	Rs-E47 termite group	-1.236	-1.957	0.079	1.000
	Marinifilaceae	-1.141	-1.892	0.088	1.000
	Muribaculaceae	-1.018	-1.496	0.166	1.000
	Oscillospiraceae	0.665	1.174	0.268	1.000
	Rikenellaceae	-0.578	-0.961	1.000	1.000
	Desulfovibrionaceae	-0.138	-0.194	0.850	1.000
MIF-KO CTL vs WT+FMT WT CAC	Saccharimonadaceae	-1.282	-1.704	0.119	1.000
	Rikenellaceae	-0.863	-1.111	1.000	1.000
	Muribaculaceae	-1.010	-1.034	0.325	1.000

Comparison	Taxon	LFC	W	p-value	*q*-value
	Bacteroidaceae	0.902	0.904	0.387	1.000
	Lachnospiraceae	0.550	0.530	0.608	1.000
	Rs-E47 termite group	-0.479	-0.494	0.632	1.000
	Oscillospiraceae	0.384	0.448	0.664	1.000
	Ruminococcaceae	0.303	0.372	0.718	1.000
	Desulfovibrionaceae	0.346	0.307	0.765	1.000
	Marinifilaceae	0.106	-0.138	0.893	1.000
MIF-KO CAC vs WT+FMT WT CAC	Ruminococcaceae	3.527	4.257	0.002	0.026*
	Lachnospiraceae	2.211	4.045	0.002	0.034*
	Oscillospiraceae	1.742	2.820	0.018	0.285
	Desulfovibrionaceae	1.975	2.163	0.056	0.875
	Muribaculaceae	-1.431	-1.905	0.086	1.000
	Saccharimonadaceae	-1.036	-1.760	0.109	1.000
	Bacteroidaceae	-0.984	-1.138	0.282	1.000
	Rs-E47 termite group	-0.664	-0.920	0.379	1.000
	Marinifilaceae	-0.463	-0.601	0.561	1.000
	Rikenellaceae	-0.122	-0.176	1.000	1.000
WT CTL vs WT+FMT MIF-KO CAC	Lachnospiraceae	-0.960	-4.190	0.002	0.029*
	Muribaculaceae	1.429	1.775	0.106	1.000
	Bacteroidaceae	1.211	1.082	0.305	1.000
	Marinifilaceae	0.588	1.001	0.341	1.000
	Ruminococcaceae	-0.384	-0.702	0.499	1.000
	Rikenellaceae	-0.387	-0.683	1.000	1.000
	Desulfovibrionaceae	-0.348	-0.406	0.693	1.000
	Saccharimonadaceae	0.206	0.396	0.700	1.000
	Oscillospiraceae	0.092	-0.213	0.835	1.000
	Rs-E47 termite group	0.005	-0.014	0.989	1.000
WT CAC vs WT+FMT MIF-KO CAC	Ruminococcaceae	1.921	3.308	0.008	0.092[E]
	Rs-E47 termite group	-1.225	-2.564	0.028	0.442
	Bacteroidaceae	-1.407	-2.000	0.073	1.000
	Desulfovibrionaceae	-1.085	-1.703	0.119	1.000
	Muribaculaceae	0.910	1.509	0.162	1.000

Comparison	Taxon	LFC	W	p-value	*q*-value
	Oscillospiraceae	0.408	0.821	0.431	1.000
	Marinifilaceae	-0.486	-0.809	0.438	1.000
	Rikenellaceae	-0.347	-0.656	1.000	1.000
	Lachnospiraceae	-0.111	0.302	0.769	1.000
	Saccharimonadaceae	0.129	0.215	0.834	1.000
MIF-KO CTL vs WT+FMT MIF-KO CAC	Bacteroidaceae	1.125	1.207	0.255	1.000
	Muribaculaceae	0.918	0.992	0.344	1.000
	Rikenellaceae	-0.632	-0.875	1.000	1.000
	Marinifilaceae	0.549	0.715	0.491	1.000
	Desulfovibrionaceae	-0.600	-0.555	0.591	1.000
	Rs-E47 termite group	-0.468	-0.533	0.606	1.000
	Lachnospiraceae	-0.440	-0.440	0.669	1.000
	Oscillospiraceae	0.127	0.156	0.879	1.000
	Ruminococcaceae	0.019	0.023	0.982	1.000
	Saccharimonadaceae	0.003	-0.004	0.997	1.000
MIF-KO CAC vs WT+FMT MIF-KO CAC	Ruminococcaceae	3.243	3.889	0.003	0.041*
	Lachnospiraceae	1.221	2.592	0.027	0.340
	Oscillospiraceae	1.485	2.676	0.023	0.341
	Desulfovibrionaceae	1.029	1.202	0.257	1.000
	Rs-E47 termite group	-0.653	-1.103	0.296	1.000
	Bacteroidaceae	-0.762	-0.967	0.356	1.000
	Muribaculaceae	0.497	0.729	0.483	1.000
	Saccharimonadaceae	0.243	0.364	0.723	1.000
	Marinifilaceae	-0.192	0.249	0.808	1.000
	Rikenellaceae	-0.109	0.172	1.000	1.000
WT CTL vs WT CAC	Ruminococcaceae	-2.305	-4.169	0.002	0.028*
	Lachnospiraceae	-1.071	-3.712	0.004	0.055[E]
	Rs-E47 termite group	1.220	2.926	0.015	0.252
	Bacteroidaceae	2.618	2.244	0.049	0.763
	Marinifilaceae	1.074	1.978	0.076	1.000
	Oscillospiraceae	-0.500	-1.070	0.310	1.000
	Desulfovibrionaceae	0.737	0.789	0.448	1.000

Comparison	Taxon	LFC	W	p-value	*q*-value
	Muribaculaceae	0.518	0.594	0.566	1.000
	Saccharimonadaceae	0.077	0.192	0.851	1.000
	Rikenellaceae	0.039	-0.062	1.000	1.000
MIF-KO CTL vs MIF-KO CAC	Ruminococcaceae	-3.224	-3.166	0.010	0.107
	Bacteroidaceae	1.886	1.797	0.102	1.000
	Lachnospiraceae	-1.661	-1.571	0.147	1.000
	Oscillospiraceae	-1.358	-1.564	0.149	1.000
	Desulfovibrionaceae	-1.629	-1.276	0.231	1.000
	Rikenellaceae	-0.741	-0.873	1.000	1.000
	Marinifilaceae	0.357	0.407	0.692	1.000
	Muribaculaceae	0.420	0.406	0.693	1.000
	Saccharimonadaceae	0.246	-0.307	0.765	1.000
	Rs-E47 termite group	0.186	0.193	0.851	1.000
WT+FMT WT CAC vs WT+FMT MIF-KO CAC	Muribaculaceae	1.928	3.266	0.008	0.141
	Lachnospiraceae	-0.991	-2.322	0.043	0.455
	Saccharimonadaceae	1.279	2.105	0.062	0.903
	Desulfovibrionaceae	-0.947	-1.550	0.152	1.000
	Marinifilaceae	0.655	1.018	0.333	1.000
	Ruminococcaceae	-0.284	-0.501	0.627	1.000
	Oscillospiraceae	-0.257	-0.479	0.642	1.000
	Rikenellaceae	0.231	0.434	1.000	1.000
	Bacteroidaceae	0.222	0.310	0.763	1.000
	Rs-E47 termite group	-0.011	0.018	0.986	1.000
WT CAC vs MIF-KO CAC	Lachnospiraceae	-1.109	-2.207	0.052	0.501
	Desulfovibrionaceae	-2.114	-2.270	0.047	0.776
	Oscillospiraceae	-1.077	-1.845	0.095	1.000
	Ruminococcaceae	-1.322	-1.578	0.146	1.000
	Rs-E47 termite group	-0.572	-0.922	0.378	1.000
	Marinifilaceae	-0.678	-0.921	0.379	1.000
	Bacteroidaceae	-0.645	-0.756	0.467	1.000
	Rikenellaceae	-0.456	-0.659	1.000	1.000
	Muribaculaceae	0.413	0.543	0.599	1.000

Comparison	Taxon	LFC	W	p-value	*q*-value
	Saccharimonadaceae	0.114	-0.197	0.848	1.000
WT CTL vs MIF-KO CTL	Rs-E47 termite group	0.463	0.547	0.596	1.000
	Lachnospiraceae	-0.520	-0.534	0.605	1.000
	Ruminococcaceae	-0.403	-0.503	0.626	1.000
	Muribaculaceae	0.511	0.456	0.658	1.000
	Rikenellaceae	0.245	0.306	1.000	1.000
	Saccharimonadaceae	0.209	0.306	0.766	1.000
	Oscillospiraceae	-0.219	-0.275	0.789	1.000
	Desulfovibrionaceae	-0.252	0.197	0.848	1.000
	Bacteroidaceae	-0.087	0.066	0.949	1.000
	Marinifilaceae	-0.039	0.054	0.958	1.000

* Significant (q < 0.05), [E] Exploratory (0.05 <= q < 0.10)

Supplementary Table S3C. Genus-level ANCOM-BC2 output including W statistics.
Detailed genus-level output for the selected ANCOM-BC2 comparisons. These results were used to support the revised genus-level interpretation and to avoid treating q-values above 0.05 as statistically significant.

Comparison	Taxon	LFC	W	p-value	q-value
WT CTL vs WT+FMT WT CAC	*Rikenella*	-1.812	-3.697	0.004	0.079[E]
	Candidatus Saccharimonas	-1.200	-3.017	0.013	0.209
	Acutalibacter	1.567	1.775	0.106	1.000
	Muribaculum	0.708	1.631	0.134	1.000
	Rikenellaceae RC9 gut group	-0.942	-1.156	0.275	1.000
	Bacteroides	0.862	0.717	0.490	1.000
	Colidextribacter	0.370	0.684	1.000	1.000
	Oscillibacter	-0.287	-0.633	1.000	1.000
	Alistipes	-0.315	-0.396	0.701	1.000
	Intestinimonas	-0.230	-0.357	0.728	1.000
	Odoribacter	-0.193	-0.339	0.741	1.000
	Lachnoclostridium	0.125	0.275	0.789	1.000
	GCA-900066575	0.027	0.076	0.941	1.000
	Lachnospiraceae NK4A136 group	0.025	0.053	1.000	1.000
WT CAC vs WT+FMT WT CAC	*Rikenellaceae RC9 gut group*	-3.128	-4.438	0.001	0.023*

Comparison	Taxon	LFC	W	p-value	q-value
	Candidatus Saccharimonas	-2.476	-4.287	0.002	0.029*
	Odoribacter	-2.467	-3.726	0.004	0.067^E
	Bacteroides	-2.955	-3.586	0.005	0.080^E
	Muribaculum	-1.461	-2.562	0.028	0.370
	Rikenella	-1.562	-2.395	0.038	0.605
	Alistipes	-1.350	-1.897	0.087	1.000
	Acutalibacter	1.233	1.649	0.130	1.000
	GCA-900066575	0.819	-1.452	0.177	1.000
	Lachnospiraceae NK4A136 group	0.519	-0.871	1.000	1.000
	Intestinimonas	0.598	-0.871	0.404	1.000
	Oscillibacter	0.535	-0.818	1.000	1.000
	Colidextribacter	0.517	-0.793	1.000	1.000
	Lachnoclostridium	0.160	0.274	0.789	1.000
MIF-KO CTL vs WT+FMT WT CAC	*Rikenella*	-1.677	-2.798	0.019	0.322
	Candidatus Saccharimonas	-1.430	-2.617	0.026	0.337
	Alistipes	-0.996	-1.486	0.168	1.000
	Lachnoclostridium	1.255	1.433	0.182	1.000
	Muribaculum	1.049	1.305	0.221	1.000
	GCA-900066575	-0.707	-1.270	0.233	1.000
	Intestinimonas	0.947	1.098	0.298	1.000
	Bacteroides	0.753	0.849	0.416	1.000
	Acutalibacter	0.409	0.602	0.560	1.000
	Lachnospiraceae NK4A136 group	0.558	0.595	1.000	1.000
	Colidextribacter	0.362	0.578	1.000	1.000
	Odoribacter	-0.255	-0.387	0.707	1.000
	Oscillibacter	0.119	0.221	1.000	1.000
	Rikenellaceae RC9 gut group	-0.032	-0.042	0.967	1.000
MIF-KO CAC vs WT+FMT WT CAC	*Candidatus Saccharimonas*	-3.162	-5.350	0.000	0.006*
	Rikenellaceae RC9 gut group	-3.522	-5.111	0.000	0.009*
	Bacteroides	-3.110	-3.912	0.003	0.053^E
	Odoribacter	-2.589	-3.642	0.005	0.073^E
	Muribaculum	-2.554	-3.157	0.010	0.159

Comparison	Taxon	LFC	W	p-value	q-value
	Acutalibacter	1.978	2.758	0.020	0.386
	Alistipes	-1.653	-2.417	0.036	0.656
	Rikenella	-1.524	-2.051	0.067	1.000
	Lachnoclostridium	0.288	0.438	0.671	1.000
	GCA-900066575	0.164	0.311	0.762	1.000
	Lachnospiraceae NK4A136 group	0.180	-0.311	1.000	1.000
	Oscillibacter	0.157	-0.201	1.000	1.000
	Colidextribacter	0.067	-0.102	1.000	1.000
	Intestinimonas	0.040	-0.061	0.952	1.000
WT CTL vs WT+FMT MIF-KO CAC	*Muribaculum*	1.735	3.178	0.010	0.159
	Lachnoclostridium	-1.363	-2.694	0.023	0.430
	Intestinimonas	-1.194	-1.963	0.078	1.000
	Odoribacter	0.758	1.176	0.267	1.000
	Bacteroides	1.382	1.120	0.289	1.000
	Lachnospiraceae NK4A136 group	-0.579	-1.111	1.000	1.000
	Rikenellaceae RC9 gut group	-1.060	-1.032	0.326	1.000
	Oscillibacter	0.334	-0.685	1.000	1.000
	Candidatus Saccharimonas	0.376	0.654	0.528	1.000
	Rikenella	0.285	0.645	0.534	1.000
	Alistipes	-0.471	-0.566	0.584	1.000
	Acutalibacter	0.144	0.155	0.880	1.000
	Colidextribacter	0.050	0.105	1.000	1.000
	GCA-900066575	0.028	-0.070	0.946	1.000
WT CAC vs WT+FMT MIF-KO CAC	*Rikenellaceae RC9 gut group*	-3.245	-3.446	0.006	0.088[E]
	Bacteroides	-2.436	-2.805	0.019	0.225
	Intestinimonas	-1.562	-2.394	0.038	0.720
	Odoribacter	-1.515	-2.082	0.064	0.787
	Lachnoclostridium	1.328	-2.127	0.059	1.000
	Alistipes	-1.506	-2.004	0.073	1.000
	Lachnospiraceae NK4A136 group	1.123	-1.790	1.000	1.000
	GCA-900066575	0.874	-1.475	0.171	1.000
	Colidextribacter	0.837	-1.396	1.000	1.000

Comparison	Taxon	LFC	W	p-value	q-value
	Candidatus Saccharimonas	0.900	-1.265	0.235	1.000
	Rikenella	0.535	0.868	0.406	1.000
	Oscillibacter	0.581	-0.858	1.000	1.000
	Muribaculum	0.434	-0.657	0.526	1.000
	Acutalibacter	0.189	-0.236	0.818	1.000
MIF-KO CTL vs WT+FMT MIF-KO CAC	*Muribaculum*	2.077	2.387	0.038	0.461
	Alistipes	-1.152	-1.617	0.137	1.000
	Bacteroides	1.273	1.371	0.200	1.000
	Acutalibacter	-1.013	-1.368	0.201	1.000
	GCA-900066575	-0.762	-1.301	0.222	1.000
	Odoribacter	0.697	0.962	0.359	1.000
	Rikenella	0.420	0.749	0.471	1.000
	Lachnoclostridium	-0.233	-0.258	0.801	1.000
	Candidatus Saccharimonas	0.145	0.211	0.837	1.000
	Rikenellaceae RC9 gut group	0.150	-0.153	0.882	1.000
	Oscillibacter	0.073	0.128	1.000	1.000
	Colidextribacter	0.042	0.073	1.000	1.000
	Lachnospiraceae NK4A136 group	-0.046	-0.047	1.000	1.000
	Intestinimonas	0.017	-0.020	0.984	1.000
MIF-KO CAC vs WT+FMT MIF-KO CAC	*Rikenellaceae RC9 gut group*	-3.640	-3.913	0.003	0.047*
	Bacteroides	-2.591	-3.081	0.012	0.164
	Candidatus Saccharimonas	1.586	-2.195	0.053	0.586
	Alistipes	-1.809	-2.494	0.032	0.607
	Odoribacter	-1.637	-2.119	0.060	0.787
	Muribaculum	-1.527	-1.746	0.111	1.000
	Lachnoclostridium	1.201	-1.730	0.114	1.000
	Intestinimonas	1.004	-1.633	0.133	1.000
	Lachnospiraceae NK4A136 group	0.784	-1.280	1.000	1.000
	Rikenella	0.573	0.804	0.440	1.000
	Acutalibacter	0.556	0.717	0.490	1.000
	Colidextribacter	0.386	-0.644	1.000	1.000
	Oscillibacter	0.204	-0.255	1.000	1.000

Comparison	Taxon	LFC	W	p-value	q-value
	GCA-900066575	0.109	0.196	0.848	1.000
WT CTL vs WT CAC	*Muribaculum*	2.169	4.213	0.002	0.033*
	Odoribacter	2.274	4.018	0.002	0.047*
	Bacteroides	3.818	3.044	0.012	0.164
	Candidatus Saccharimonas	1.276	2.772	0.020	0.278
	Rikenellaceae RC9 gut group	2.185	2.498	0.032	0.381
	GCA-900066575	-0.846	1.763	0.108	1.000
	Colidextribacter	-0.887	1.759	1.000	1.000
	Alistipes	-1.035	1.234	0.246	1.000
	Lachnospiraceae NK4A136 group	-0.544	0.954	1.000	1.000
	Intestinimonas	-0.368	0.553	0.592	1.000
	Rikenella	-0.250	-0.461	0.655	1.000
	Oscillibacter	-0.248	0.406	1.000	1.000
	Acutalibacter	-0.334	0.347	0.736	1.000
	Lachnoclostridium	-0.035	-0.073	0.943	1.000
MIF-KO CTL vs MIF-KO CAC	*Rikenellaceae RC9 gut group*	3.490	4.322	0.002	0.026*
	Bacteroides	3.864	4.151	0.002	0.038*
	Muribaculum	3.604	3.511	0.006	0.096[E]
	Odoribacter	2.334	3.313	0.008	0.110
	Candidatus Saccharimonas	1.731	2.853	0.017	0.259
	Acutalibacter	-1.569	-2.095	0.063	1.000
	GCA-900066575	-0.871	-1.422	0.185	1.000
	Intestinimonas	-0.987	1.161	0.273	1.000
	Lachnoclostridium	-0.967	1.029	0.328	1.000
	Alistipes	-0.657	0.945	0.367	1.000
	Lachnospiraceae NK4A136 group	-0.738	0.755	1.000	1.000
	Colidextribacter	-0.428	0.720	1.000	1.000
	Oscillibacter	-0.277	0.346	1.000	1.000
	Rikenella	-0.153	-0.208	0.839	1.000
WT+FMT WT CAC vs WT+FMT MIF-KO CAC	*Rikenella*	2.097	3.675	0.004	0.079[E]
	Candidatus Saccharimonas	1.576	2.343	0.041	0.498
	Lachnoclostridium	-1.489	-2.462	0.034	0.608

Comparison	Taxon	LFC	W	p-value	q-value
	Acutalibacter	-1.423	-2.007	0.073	1.000
	Muribaculum	1.027	1.716	0.117	1.000
	Intestinimonas	-0.964	-1.526	0.158	1.000
	Odoribacter	0.952	1.302	0.222	1.000
	Lachnospiraceae NK4A136 group	-0.604	-1.103	1.000	1.000
	Bacteroides	0.519	0.655	0.527	1.000
	Colidextribacter	-0.320	-0.507	1.000	1.000
	Alistipes	0.156	-0.222	0.829	1.000
	Rikenellaceae RC9 gut group	0.118	-0.133	0.897	1.000
	GCA-900066575	0.055	-0.110	0.914	1.000
	Oscillibacter	0.047	-0.086	1.000	1.000
WT CAC vs MIF-KO CAC	*GCA-900066575*	-0.983	-1.587	0.143	1.000
	Muribaculum	1.093	1.278	0.230	1.000
	Candidatus Saccharimonas	0.686	1.081	0.305	1.000
	Acutalibacter	-0.745	-0.918	0.380	1.000
	Intestinimonas	-0.558	-0.832	0.425	1.000
	Colidextribacter	-0.451	-0.724	1.000	1.000
	Rikenellaceae RC9 gut group	0.395	0.520	0.614	1.000
	Lachnospiraceae NK4A136 group	-0.339	-0.517	1.000	1.000
	Oscillibacter	-0.378	-0.429	1.000	1.000
	Alistipes	0.303	0.413	0.689	1.000
	Lachnoclostridium	-0.128	-0.189	0.854	1.000
	Bacteroides	0.155	0.178	0.862	1.000
	Odoribacter	0.122	0.172	0.867	1.000
	Rikenella	-0.038	-0.048	0.962	1.000
WT CTL vs MIF-KO CTL	*GCA-900066575*	0.734	1.558	0.150	1.000
	Lachnoclostridium	-1.130	-1.394	0.194	1.000
	Intestinimonas	-1.177	-1.392	0.194	1.000
	Acutalibacter	1.157	1.274	0.231	1.000
	Rikenellaceae RC9 gut group	-0.910	-0.992	0.344	1.000
	Alistipes	0.682	0.847	0.417	1.000
	Oscillibacter	-0.407	-0.837	1.000	1.000

Comparison	Taxon	LFC	W	p-value	q-value
	Lachnospiraceae NK4A136 group	-0.533	-0.577	1.000	1.000
	Candidatus Saccharimonas	0.231	0.549	0.595	1.000
	Muribaculum	0.342	-0.446	0.665	1.000
	Rikenella	0.135	-0.282	0.784	1.000
	Odoribacter	0.062	0.110	0.915	1.000
	Bacteroides	-0.109	0.084	0.935	1.000
	Colidextribacter	0.008	0.017	1.000	1.000

* Significant (q < 0.05), [E] Exploratory (0.05 <= q < 0.10)

Supplementary Table S4. Summary of the exploratory ASV-level ANCOM-BC2 analysis.

An additional ASV-level ANCOM-BC2 analysis was performed without taxonomic agglomeration to evaluate whether genus-level comparisons could mask subtler differences. Because species-level assignment from the 16S rRNA V3-V4 region is limited and varies across bacterial taxa, these results were interpreted only at the ASV level with the corresponding taxonomic assignment. The ASV-level results are summarized here to document the additional exploratory analysis while avoiding overinterpretation of non-significant contrasts.

Supplementary Table S4A. Summary of ASV-level ANCOM-BC2 results by comparison.

This table summarizes the ASV-level analysis by contrast, including the number of ASVs tested, the number of ASVs meeting q < 0.05, and the minimum q-value observed in each comparison. The direct WT CAC vs. MIF-KO CAC comparison and the WT+FMT WT CAC vs. WT+FMT MIF-KO CAC comparison did not show significant ASV-level differences after multiple-testing correction.

Comparison	Tested ASVs	ASVs with q-value	Significant ASVs q < 0.05	Exploratory ASVs 0.05 <= q < 0.10	Minimum q-value
WT CTL vs WT+FMT WT CAC	7	7	0	0	0.143
WT CAC vs WT+FMT WT CAC	7	7	0	0	0.115
MIF-KO CTL vs WT+FMT WT CAC	7	7	0	0	1.000
MIF-KO CAC vs WT+FMT WT CAC	7	7	0	0	0.328
WT CTL vs WT+FMT MIF-KO CAC	7	7	1	0	0.029
WT CAC vs WT+FMT MIF-KO CAC	7	7	1	0	0.044
MIF-KO CTL vs WT+FMT MIF-KO CAC	7	7	0	0	1.000
MIF-KO CAC vs WT+FMT MIF-KO CAC	7	7	0	0	0.102
WT CTL vs WT CAC	7	7	2	0	0.009
MIF-KO CTL vs MIF-KO CAC	7	7	0	0	1.000
WT+FMT WT CAC vs WT+FMT MIF-KO CAC	7	7	0	0	0.409
WT CAC vs MIF-KO CAC	7	7	0	0	1.000
WT CTL vs MIF-KO CTL	7	7	0	0	0.918

Supplementary Table S4B. Significant ASVs detected in the exploratory ASV-level ANCOM-BC2 analysis.

Only ASVs meeting $q < 0.05$ are shown in detail. These significant ASVs were detected in a limited number of comparisons and did not include the direct WT CAC vs. MIF-KO CAC or WT+FMT WT CAC vs. WT+FMT MIF-KO CAC contrasts. Therefore, the ASV-level analysis was considered exploratory and was used to support a conservative interpretation of the genus-level findings.

Comparison	ASV	Family	Genus	Species	LFC	W	p-value	*q*-value
WT CTL vs WT+FMT MIF-KO CAC	ASV_0062	Muribaculaceae	Unassigned	Unassigned	1.116	4.155	0.002	0.029
WT CAC vs WT+FMT MIF-KO CAC	ASV_0020	Rs-E47 termite group	Unassigned	Unassigned	-1.828	-3.837	0.003	0.044
WT CTL vs WT CAC	ASV_0020	Rs-E47 termite group	Unassigned	Unassigned	1.727	4.924	0.001	0.009
WT CTL vs WT CAC	ASV_0002	Marinifilaceae	Odoribacter	Unassigned	1.906	4.479	0.001	0.018

Supplementary Table S5. Major findings supporting a role for MIF in modulating the gut microbiota during colitis-associated colorectal cancer.

Results overview	Principal finding	Key supporting evidence	Interpretation
Protumorigenic effect of MIF deficiency-associated microbiota	MIF-KO mice developed a more severe CAC phenotype and a significantly higher tumor burden than WT mice	MIF-KO CAC mice showed earlier clinical manifestations and significantly increased tumor numbers relative to WT CAC mice	MIF deficiency is associated with enhanced tumor progression in AOM/DSS-induced CAC
FMT experiments	The MIF-KO-derived microbiota transferred a protumorigenic phenotype to WT recipients	WT recipients colonized with MIF-KO microbiota exhibited greater weight loss, shorter colons, and higher tumor burden than recipients colonized with WT microbiota	The microbiota associated with MIF deficiency functionally contributes to tumor promotion
Sequence quality control	Sequence processing generated a robust dataset for downstream microbiome analyses	Overall chimera rate was 3.57%, with a median of 14,738 non-chimeric reads/sample	Sequencing depth and quality support the reliability of the ecological analyses
α-diversity	No significant pairwise differences in alpha diversity were retained after multiple-testing correction	Chao1, observed ASVs, Shannon, and Simpson indices showed global variation, but no corrected pairwise contrasts remained significant	Tumor progression was not associated with a major loss of overall microbial richness or diversity
β-diversity	Microbial community structure differed across groups, particularly under weighted UniFrac analysis	PCoA based on Bray–Curtis and weighted UniFrac showed separation among controls, CAC groups, and FMT recipient groups	The relevant microbiota-associated changes were primarily compositional and phylogenetically structured
Phylum-level alterations	CAC induced consistent phylum-level dysbiosis across tumor-bearing groups	Increased Bacteroidota, Thermodesulfobacteroidota, and Patescibacteria, together with reduced Bacillota and Campylobacterota	CAC is associated with broad ecological restructuring linked to inflammatory and tumor-associated conditions
WT CAC versus MIF-KO CAC	MIF-KO CAC mice exhibited a more pronounced dysbiotic profile than WT CAC mice	MIF-KO CAC mice showed greater expansion of Bacteroidota and stronger reduction in Bacillota than WT CAC mice	The absence of MIF exacerbates tumor-associated microbial remodeling
Family-level alterations	Families linked to intestinal homeostasis decreased, whereas inflammation-associated families expanded during CAC	Lower relative abundance of Lachnospiraceae and Oscillospiraceae, with increases in Muribaculaceae, Bacteroidaceae, and Marinifilaceae	CAC progression is accompanied by selective restructuring of key bacterial families involved in intestinal ecological balance
Genus-level alterations	Several genera were associated with the tumor-related microbial shift	Reduced *Lachnospiraceae NK4A136* group, *Alistipes*, and *Lachnoclostridium*; increased *Bacteroides*, *Odoribacter*, and *Rikenellaceae RC9* gut group	CAC progression is linked to enrichment of genera commonly associated with inflammation, mucus degradation, or dysbiosis
FMT recipient profiles	WT-derived microbiota showed greater ecological resilience than MIF-KO-derived microbiota	WT+FMT WT CAC remained closer to WT control-like profiles, whereas WT+FMT MIF-KO CAC displayed a more dysbiotic configuration, including increased Bacteroides	Donor microbiota origin strongly influences microbial reassembly and tumor-related outcome after FMT
ANCOM-BC2 family	Only limited family-level	Significant decrease in Ruminococcaceae in WT CTL vs.	Family-level differences were

analysis	differential abundance was detected	WT CAC and decrease in Lachnospiraceae in WT CTL vs. WT+FMT MIF-KO CAC	relatively modest despite clear phenotypic differences
ANCOM-BC2 genus analysis	Genus-level biomarkers were more informative than family-level markers	Muribaculum and Odoribacter were enriched in WT CAC, whereas Bacteroides and Rikenellaceae RC9 gut group were enriched in MIF-KO CAC	The most discriminative microbial signatures associated with CAC and MIF deficiency occur at the genus level
Overall conclusion	MIF acts as a host modulator of the microbiota–tumor axis in CAC	MIF deficiency was associated with a more protumorigenic microbiota that could be functionally transferred by FMT	MIF may constrain microbial configurations or functions that favor inflammation-associated colorectal carcinogenesis

Footnote: Data were derived from phenotypic analyses of AOM/DSS-induced colitis-associated colorectal cancer (CAC), fecal microbiota transplantation (FMT) experiments, 16S rRNA gene sequencing, α- and β-diversity analyses, taxonomic profiling, and ANCOM-BC2 differential abundance testing. WT, wild type; MIF, macrophage migration inhibitory factor; MIF-KO, MIF-deficient; CAC, colitis-associated colorectal cancer; FMT, fecal microbiota transplantation; ASV, amplicon sequence variant.
